# Neuropathic pain relief and altered brain networks after dorsal root entry zone microcoagulation in patients with spinal cord injury

**DOI:** 10.1093/braincomms/fcae411

**Published:** 2024-11-21

**Authors:** Scott Falci, Leslie Morse, Jeffrey Berliner, Mario Murakami, Abigail Welch, David Barnkow, Nguyen Nguyen, Ricardo Battaglino, Clas Linnman

**Affiliations:** Department of Neurosurgery, Swedish Medical Center, Englewood, CO 80113, USA; Department of PM&R, University of Miami Miller School of Medicine, Miami, FL 33136, USA; Research Department, Craig Hospital, Englewood, CO 80113, USA; Spaulding Neuroimaging Laboratory, Spaulding Rehabilitation Hospital, Harvard Medical School, Boston, MA 02129, USA; Research Department, Craig Hospital, Englewood, CO 80113, USA; Medsurant Health, LLC, Englewood, CO 80113, USA; Department of Rehabilitation Medicine, University of Minnesota, Minneapolis, MN 55455, USA; Department of PM&R, University of Miami Miller School of Medicine, Miami, FL 33136, USA; Spaulding Neuroimaging Laboratory, Spaulding Rehabilitation Hospital, Harvard Medical School, Boston, MA 02129, USA

**Keywords:** spinal cord injury, dorsal root entry zone, neuropathic pain, sympathetic, fMRI

## Abstract

Spinal cord injury (SCI) below-level neuropathic pain is a difficult condition to treat both pharmacologically and surgically. Successful treatment using surgically created lesions of the spinal cord dorsal root entry zone (DREZ), guided by intramedullary monitoring of neuronal electrical hyperactivity, has shown that DREZs both cephalad and caudal to the level of injury can be the primary generators of SCI below-level pain. Below-level pain perception follows a unique somatotopic map of DREZ pain generators, and neuronal transmission to brain pain centres can occur primarily through sympathetic nervous system (SNS) pathways. This study evaluated changes in brain resting-state and task-based functional magnetic resonance imaging responses before and after neuroelectrically guided DREZ microcoagulation surgery. Eight persons with clinically complete SCI who suffered chronic, severe and unrelenting below-level neuropathic pain refractory to all pharmacological management were investigated before and after the surgical intervention. Baseline differences between DREZ subjects, group-matched low pain SCI and healthy controls were observed in medial primary somatosensory and motor cortex connectivity to the hippocampus, amygdala and medial prefrontal cortex. The DREZ surgery led to short-term (12 days) almost complete pain relief in all participants and long-term (1+ year) pain relief in all participants receiving DREZ lesioning both cephalad and caudal to the level of injury (six out of eight participants). Follow-up 12 days post-operatively indicated that DREZ surgery normalized prior negative functional coupling between primary sensory (S1) and motor (M1) cortices to the hippocampus, amygdala and the medial prefrontal cortex, increased M1 to putamen and amygdala connectivity and decreased limbic to cerebellar connectivity. DREZ hyperactivity was found both cephalad and caudal to the level of injury. The regional distribution of hyperactive regions corresponded not to classical dermatomes but rather mapped on to intermediolateral (IML) cell column end organ innervation of body regions of below-level pain perception, consistent with a non-classical SNS-mediated somatotopic map of DREZ below-level pain generators. The results indicate that neuroelectrically guided DREZ microcoagulation alters a medial prefrontal–somatosensory–limbic network that is separate from classical pain pathways. This provides further evidence that below-level SCI pain originates in hyperactive DREZs and can be relayed to the brain via the SNS.

## Teaser

Falci *et al.* report that neuroelectrically guided dorsal root entry zone ablation can result in complete relief of chronic neuropathic pain after spinal cord injury. Altered limbic–somatosensory–prefrontal resting-state connectivity was also observed. The surgical intervention was done primarily below the spinal lesion, indicating pain transmission via sympathetic afferents.

## Graphical Abstract

**Graphical Abstract:**
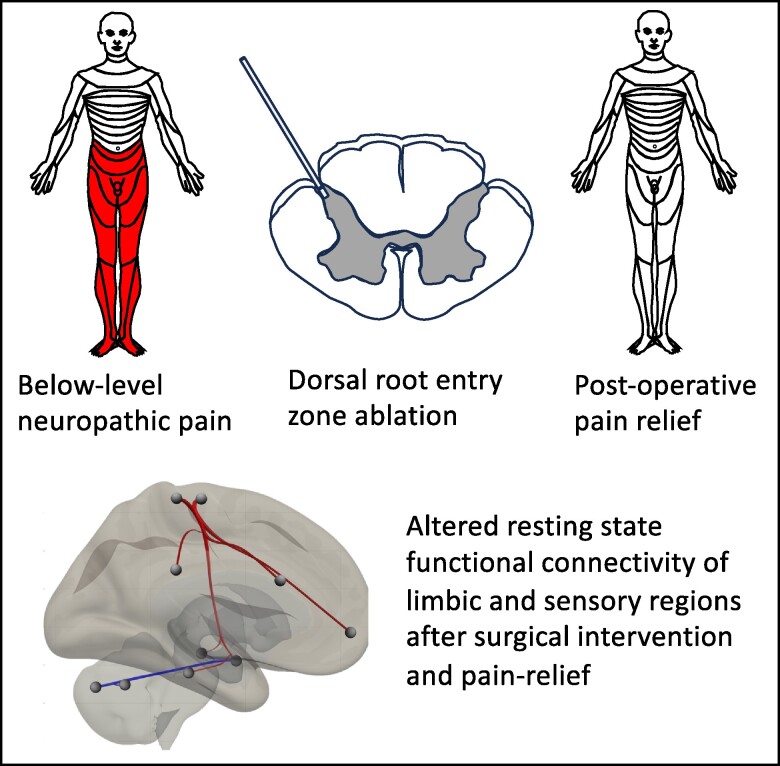

## Introduction

Neuropathic pain is perhaps the most disabling of sequelae to spinal cord injury (SCI), perceived at the level of injury (point prevalence ≈ 19%), and/or below the level of injury (point prevalence ≈ 27%).^1^ At-level and below-level pains are experienced in regions of anaesthesia or hypaesthesia and are commonly described as ‘sharp, burning, electrical, stabbing and/or pins and needles’. Our prior work has shown that SCI can induce neuronal hyperexcitability in dorsal root entry zones (DREZs) cephalad and caudal to the level of injury, specifically in posterior grey matter regions of the first synapse of peripheral C-fibre and a-delta fibre sensory neurons (Rexed laminae 1–3). Identification and microcoagulation of the hyperactive DREZ regions can abolish both at-level and below-level pains.^2^ In patients with SCI and complete spinal cord transections at the injury site, DREZ ablation surgery performed *only* caudal to the transections led to excellent below-level pain relief.^3^ This indicates that signals from hyperactive DREZ can be routed around a spinal lesion via the sympathetic nervous system (SNS) to the brain to create the experience of pain. These DREZ ‘pain generators’ also exhibit a novel somatotopic map that corresponds with IML cell column end organ innervation of body regions of perceived pain. For example, DREZ hyperactivity at T7–T10 can lead to rectal and genital pain, in regions that are classically innervated by sacral nerves.^3,4^

Here, we used functional magnetic resonance imaging (fMRI) to determine brain functional connectivity patterns and task-induced responses in patients with severe below-level neuropathic pain before and after DREZ microcoagulation. Participants were further compared with a matched group of persons with SCI with low levels of pain [low pain (LP)] and to persons with neither pain nor SCI [health control (HC)].

## Materials and methods

### Participants

Nine participants with SCI were enrolled in an observational study assessing surgical treatment for severe neuropathic pain, henceforth called the pre-DREZ group. Individuals were eligible for the study if they had 9/10 pain or greater and were willing and scheduled to undergo DREZ microcoagulation procedure to treat their pain. One participant dropped out of the study and was not included in the analyses. We also included a sample of baseline data from eight age-matched participants with SCI and LP enrolled in an ongoing clinical trial (ClinicalTrials.gov Identifier: NCT02533713) and 12 non-injured controls (HC) (see Table 1).

**Table 1:** Study sample demographic and pain ratings at baseline

Group	Sex	Age	Level	Years since SCI	Pain at worst in last 7 days	Opioid medication
DREZ	M	70	T10 A	7	8	N
DREZ	M	30	T8 A	3	9	Y
DREZ	M	60	T12 A	30	8	N
DREZ	M	40	T10 A	9	8	N
DREZ	M	50	T12 A	33	10	N
DREZ	M	60	T10 A	5	10	N
DREZ	F	50	T10 A	5	9	Y
DREZ	M	20	T10 A	3	10	Y
						
Low pain SCI	M	40	T6 A	17	0	N
Low pain SCI	M	20	T10 A	4	4	Y
Low pain SCI	M	40	T6 A	5	3	N
Low pain SCI	M	30	T9 A	4	1	N
Low pain SCI	M	40	T7 A	19	4	N
Low pain SCI	M	30	T6 A	14	3	Y
Low pain SCI	M	40	T12 A	17	5	Y
Low pain SCI	M	60	T11 A	3	2	N
						
HC	M	60	-	-	2	N
HC	M	30	-	-	0	N
HC	M	30	-	-	2	N
HC	M	50	-	-	0	N
HC	M	30	-	-	2	N
HC	M	50	-	-	0	N
HC	M	40	-	-	3	N
HC	M	30	-	-	0	N
HC	M	30	-	-	1	N
HC	M	30	-	-	0	N
HC	F	30	-	-	1	N
HC	F	40	-	-	-	N

Age is rounded to nearest decade.

DREZ, dorsal root entry zone; HC, healthy controls; SCI, spinal cord injury.

### Pre-operative assessments

All pre-DREZ underwent clinical pre-operative plain radiography, computed tomography and MRI to evaluate the spine and spinal cord. All pre-DREZ had tried multiple pharmacological treatments for neuropathic pain control, including antidepressant, antiseizure and opioid medications. These medications were considered ineffective or provided inadequate neuropathic pain relief, yet all were fearful of discontinuation. Prior to surgery, two patients indicated that they did not want the DREZ procedure performed cephalad to the level of injury for fear of compromising upper extremity function. These participants were informed that surgery might not eradicate all regions of DREZ hyperactivity and that significant or long-lasting pain relief may not be achieved if neuroelectrical hyperactivity existing cephalad to the level of injury was contributory to their pain.

### Clinical outcome measures

The regional distribution of current pain was evaluated before and after the surgical intervention by means of a 0–10 verbal scale with 10 indicating near suicidal-level pain (see Table 2). One to 3 days prior to surgery and 18–88 days after surgery (average 31 days), pre-DREZ was assessed using the Patient Health Questionnaire 9 and the Beck Depression Inventory to measure depressive symptoms, the Short-form McGill Pain Questionnaire, the Brief Pain Inventory, the Pain Catastrophizing Scale and the Tampa Scale of Kinesiophobia (see Table 3). ^5-11^

**Table 2:** Pain and depression rating scales in DREZ subjects before and after intervention (1-month follow-up)

Scale	Baseline (SD)	Post-op (SD)	*P*-value	*n*
PHQ-9	17.1 (6.3)	6.1 (5.4)	0.02	7
BDI	29.3 (15.2)	8.3 (4.4)	0.03	6
McGill_total_	25.9 (13.1)	7.8 (7.9)	0.02	8
McGill_sensory_	18.9 (8.6)	5.6 (6.4)	0.02	8
McGill_emotional_	7.0 (4.5)	2.1 (2.0)	0.01	8
BPI	40.8 (16.6)	15.8 (14.9)	0.01	8
PCS_total_	35.5 (12.0)	10.6 (11.9)	0.005	8
PCS_rumination_	12.4 (4.1)	4.9 (6.3)	0.02	8
PCS_magnification_	5.9 (3.7)	1.4 (1.8)	0.005	8
PCS_helplessness_	17.3 (5.1)	4.4 (5.4)	0.005	8
TSK	41.7 (9.4)	34.5 (7.1)	0.04	6

BDI, Beck Depression Inventory; BPI, Brief Pain Inventory; McGill, shortform McGill Pain Questionnaire; PHQ-9, Patient Health Questionnaire 9; PCS, the Pain Catastrophizing Scale; TSK, the Tampa Scale of Kinesiophobia.

**Table 3:** Regional pain distribution at baseline, DREZ ablation levels and follow-up

Patient	1	2	3	4	5	7	8	9
**Region of pre-op pain**								
Groin	3	10	0	8	10	0	10	10
Quad	3	10	0	0	10	0	10	10
Ham	3	10	0	8	0	10	10	10
Calf	3	10	0	8	0	0	10	10
Shin	3	10	0	0	0	0	10	10
Foot	3	10	10	8	0	0	10	10
Rectal/groin/genitalia	0	0	8	0	10	10	10	0
Trunk	9.5	0	0	8	0	0	0	0
**DREZ levels of hyperactivity**								
T9	X	-	X	-	-	-	X	-
T10	X	-	X	X	-	X	X	X
T11	-	X	X	X	X	X	X	X
T12	-	X	X	X	X	-	-	X
L1	X	X	X	X	X	-	-	X
L2	X	X	X	X	X	-	-	X
L3	X	X	X	-	-	-	-	-
L4	X	X	X	-	-	-	-	-
L5	X	X	X	-	-	-	-	-
S1	X	X	X	-	-	-	-	-
S2	X	-	-	-	-	-	-	-
**Regional relative % pain change post-operatively**								
Groin	−100	−100^a^	-	−100	−100	-	−100	−100
Quad	−100	−100^a^	-	-	−100	-	−100	−100
Ham	−100	−100^a^	-	−100	-	−100^c^	−100	−100
Calf	−100	−100^a^	-	−100	-	-	−100	−100
Shin	−100	−100^a^	-	-	-	-	−100	−100
Foot	−100	−100^a^	−75	−100	-	-	−70	−87.5
Rectal/groin/genitalia	-	-	−40	-	−100	−100^c^	−100	-
Trunk	−100	-	-	−100^b^	-	-	-	-

X, hyperactive; -, not hyperactive.

^a^Subject experienced full return of pain at 1-year follow-up.

^b^Subject experienced complete trunk pain relief and also a new silver dollar size truncal pain in different position.

^c^Subject experienced full return of pain at 1-year follow-up.

Follow-up observation regarding pain relief was accomplished by telephone interview and outpatient evaluation at least 1 year post-operatively.

### Neuroimaging

All participants underwent structural, diffusion, resting-state and task functional MRI. The tasks were acquired with a blocked design of active finger or imagined foot tapping. Pre-DREZ underwent neuroimaging 1–2 days (average 1.4 days) prior to and 11 days (range 7–15 days) after the DREZ surgery (post-DREZ). All data were collected at Craig Hospital, Denver, CO, USA, using a 3-T Siemens Trio with a 12-channel head coil (see Supplementary material for data acquisition parameters).

### DREZ neuroelectrically guided lesioning

The methods of DREZ electrical recordings performed cephalad and caudal to the level of injury and DREZ microcoagulation performed cephalad to the level of injury used in this study were as previously described.^3^ Laminectomies were performed to expose the spinal cord at the injury site, as well as at levels cephalad and caudal to the injury. An active electrode was inserted into the specific DREZ, and spontaneous electrophysiological recordings were obtained for 1 s. Briefly, analyses were performed using a subroutine in the Cadwell Cascade software where the initial data were analysed by root mean square (expressed in microvolts), frequency and voltage in the waveform by fast Fourier transform and area under the waveform curve (expressed in microvolt milliseconds). Both root mean square analysis and area under the waveform data provided a single numerical value of the recorded neuroelectrical energy. A phenomenon that we describe as ‘spindles’ was examined by passing the initial data through a tight digital filter with a band pass of 65–100 Hz. A visual count was made of the number of spindle bursts in the 1-s recording, excluding artefacts caused by cardiac electrical activity or electrode movement. With these analyses, two distinct electrophysiological DREZ activities were found, consistent with our previous study.^3^ Those activities showing lower voltage and frequencies, smaller area under the waveform curve and fewer than three spindles were considered to be non–pain-producing DREZ activity; those showing higher voltage and frequencies, greater area under the waveform curve and three or more spindles were considered to be regions of abnormal pain-producing neuroelectrical hyperactivity. These same analyses were also performed subsequent to DREZ microcoagulation of regions of abnormal neuroelectrical hyperactivity. Examples of recordings are provided in Supplementary Fig. 2. DREZ microcoagulation caudal to the level of injury was performed using a needle-tipped electrocautery (Covidien Force FX electrosurgical generator; Valleylab, Inc.) at a setting of 10 desiccate for a 1-s pulse, with 1 ml of separation between lesions, in all DREZs in which spontaneous neuroelectrical hyperactivity was demonstrated.

### Data analysis

#### Resting-state connectivity analysis

All functional data was pre-processed and statistically analysed using the CONN Toolbox (version 21a),^12^ a cross-platform software operating under Statistical Parametric Mapping (SPM12)^13^ and MATLAB. Default parameters, including slice timing, motion correction, spatial normalization to the Montreal Neurological Institute (MNI) template, spatial smoothing with an 8-mm Gaussian kernel and high-pass temporal filtering (cut-off 128 s), were used in pre-processing. The Artifact Detection Toolbox (https://www.nitrc.org/projects/artifact_detect) was used to detect frames with excessive motion (global signal value *z* > 5, inter-scan motion > 0.9 mm), which were regressed out of the time series in CONN’s denoising pipeline in addition to white matter and cerebrospinal fluid signal using aCompCor.

We used a seed-based correlations approach with 14 regions of interest, investigating both classical pain pathways—thalamus, somatotopic sensory and motor representation areas of the lower body (medial pre-central and post-central gyri and posterior insula), the periaqueductal grey, anterior insula and anterior cingulate—as well as the central autonomic network (posterior mid-cingulate cortex, amygdala and the hippocampus).^14-19^

Regions of interest were defined in the Conn toolbox except the periaqueductal grey (defined as a 5-ml radius sphere centred at MNI 0, −30 and −24).^20^ The posterior insula lower leg representation region was derived from Bjornsdotter *et al.*^17^ The medial pre- and post-central gyri were defined as the intersection of the Conn toolbox was defined superior sensorimotor network and the pre-central or post-central gyri, respectively. Follow-up analyses of somatosensory and motor cortices are presented in the Supplementary material.

We first conducted a three-group ANOVA contrasting pre-DREZ with LP and HC and *post hoc* group *t*-tests. Second, we conducted a within group repeated measures *t*-test of the pre-DREZ to post-DREZ subjects. Connectivity values of clusters from the ANOVA and in the pre–post-DREZ analysis were extracted for visualization and interpretation.

We used a critical cluster threshold of *P* < 0.05 false discover rate (FDR) corrected for multiple comparisons for the task analyses. For the resting-state functional connectivity (rsFC) analysis, the difference surviving Bonferroni correction for 14 regions of interest, i.e. *P*_FDR_ < 0.0036, was considered significant.

#### Foot tap and finger tap

The finger tap and foot tap paradigms were modelled in SPM12^13^ using a general linear model with a boxcar regressors signifying the finger/foot tap onsets and durations (four sets of 30-s tapping blocks) convolved with the SPM12 canonical haemodynamic response function, six motion regressors and outliers removal (ART toolbox). For between-subject comparisons, an ANOVA model was used including the eight baseline pre-DREZs, the eight LPs and the 12 HCs. A repeated measure *t*-test was used within the DREZ group for analysis of finger and imagined foot tapping activations before and after surgery.

#### Structural and diffusion imaging

Cortical thickness analyses and diffusion-weighted imaging data collection, analysis and results are reported in Supplementary material.

## Results

### Participant demographics

Pre-DREZ, LP and HC did not differ with regard to sex distribution (*χ*^2^) or age (ANOVA). Injury levels (T6–T12), completeness (all American Spinal Injury Association Impairment Scale Grade A) and time since injury (3–33 years) were similar within pre-DREZ and LP. Pain at worst in the past 7 days differed significantly between the groups *F*(2,24) = 99.6, *P* = 2.4 ∗ 10^−12^. *Post hoc* tests indicated pain at worst was higher in pre-DREZ (9 out of 10 on average) compared with LP and HC, but also significantly higher in the LP group compared with HC (2.75 versus 1 on average). Notably, opioid use was similar in pre-DREZ and LP, but non-existent in the HC (Fisher exact *P* < 0.05) (see Table 1).

All pre-DREZ experienced continuous neuropathic pain with bursts in intensity occurring multiple times daily, exacerbated by any noxious stimulus to the body, such as a skin sore or urinary tract infection. Pain was experienced in regions of the body in which sensation was absent to testing (see Table 3). In clinical interviews, six of eight patients rated their worst pain experienced on a daily basis as a 10 on a scale of 1–10, with 10 considered near suicidal-level pain, one patient rated a 9.5 and one an 8.

### Pain relief and improved mood post-surgery

All eight DREZ patients achieved essentially complete relief of below-level neuropathic pain at the time of post-operative MRI. Across all painful regions (Table 3), the pain relief in impacted regions was −9.3 (range −10 to −7.5, 95% confidence interval −10 to −8.3, *P* < 0.0001) on the 0–10 Visual Analog Scale. The one patient who additionally experienced at-level pain achieved complete relief of this band of pain.

The two patients who did not undergo DREZ microcoagulation cephalad to the level of injury for fear of losing preserved function initially experienced pain relief but subsequently had essentially complete return of below-level neuropathic pain by 2 months post-operatively. Of the six patients who underwent DREZ microcoagulation of all regions of DREZ hyperactivity, five achieved complete or near-complete relief of neuropathic pain and one achieved ∼50% neuropathic pain relief at a 1-year follow-up. Six of the eight patients successfully weaned off their opioid medications, antiseizure medications and antidepressant medications. Similarly, all participants experienced significant improvements in mood, reductions in pain catastrophizing and reductions in kinesiophobia (Table 2).

### Surgery-related morbidity

All eight patients underwent pre- and post-operative sensory and motor testing. There was no loss of motor function after the intervention reported in seven of these eight patients. One patient reported some decrease in truncal control, which did not interfere with his routine daily activities. Of the six patients who underwent DREZ microcoagulation cephalad to the level of injury, all reported their sensory level ascending to the most cephalad dermatomal level of DREZ microcoagulation. In the two patients in whom DREZ microcoagulation was performed solely caudal to the level of injury, no change in sensation or sensory level was reported. There were no cases of wound infection, deep venous thrombosis, pulmonary embolus, cerebrospinal fluid leakage or death. One patient who did not undergo DREZ microcoagulation cephalad to his level of injury developed thoracolumbar spinal instability with significant canal stenosis and return of his neuropathic pain at 2 months post-operatively. He chose not to pursue corrective surgical decompression and stabilization. One patient developed a new at-level silver dollar-sized region of truncal neuropathic pain at his new sensory level post-DREZ microcoagulation, which was rated an 8 out of 10.

### Resting-state functional connectivity

**Figure 1:**
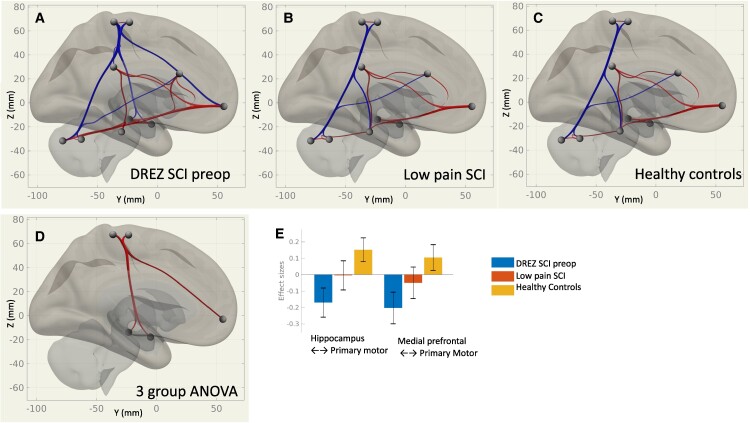
**Overview of group rsFC in the hippocampus, amygdala, primary sensory cortex, primary motor cortex (M1), medial prefrontal cortex, anterior and posterior cingulate and cerebellum.** (**A**) DREZ surgery SCI group with severe neuropathic pain at baseline. (**B**) Matched LP SCI group. (**C**) Healthy control group. Red paths indicate positive rsFC and blue negative rsFC. (**D**) Three-group ANOVA with red paths indicating higher connectivity in healthy controls. (**E**) *Post hoc* display of effect sizes for rsFC in between the left hippocampus and the medial pre-central gyrus for each group.

**Figure 2:**
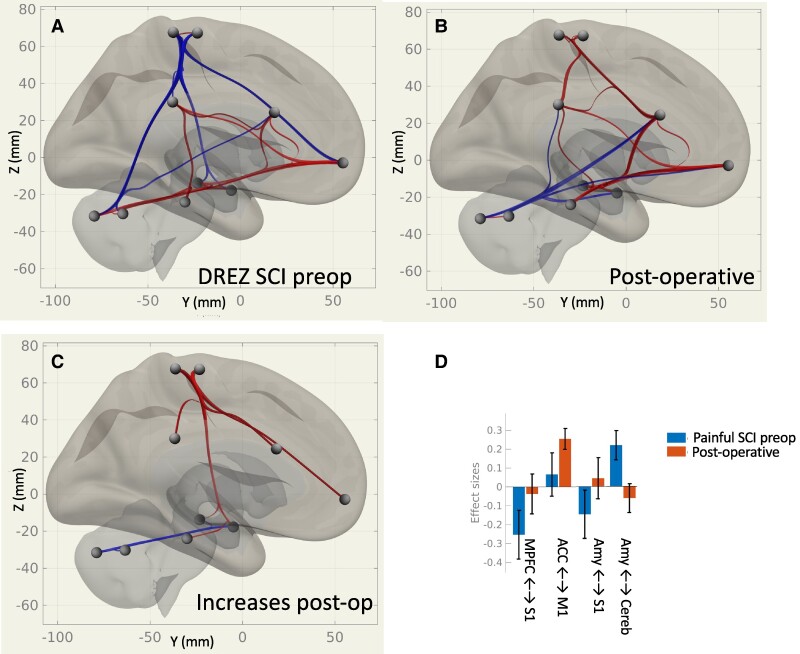
**Overview of rsFC in the DREZ SCI group before (A) and after (B) DREZ intervention on resting-state connectivity in the hippocampus, amygdala, primary sensory (S1), primary motor (M1), anterior cingulate and posterior cingulate cortex, cerebellum and periaqueductal grey.** (**C**) A repeated measures *t*-test indicates significant changes in rsFC, with red paths indicating increased rsFC after the DREZ intervention. (**D**) Effect sizes of rsFC change for some key pathways. Red paths indicate positive/increased rsFC and blue negative/decreased rsFC. ACC, anterior cingulate; PCC, posterior cingulate cortex.

We assessed differences in rsFC based on group (pre-DREZ, LP and HC) and based on intervention (pre- to post-DREZ). An overview of resting-state connectivity group and treatment effects is presented in Figs 1 and 2. The overall findings indicate an abnormal anticorrelation between the primary somatomotor cortex to the hippocampi and to the medial prefrontal cortex in pre-DREZ. This abnormal anticorrelation was ‘normalized’ after DREZ and pain relief, along with a decrease in amygdala-to-cerebellar connectivity.

**Figure 3:**
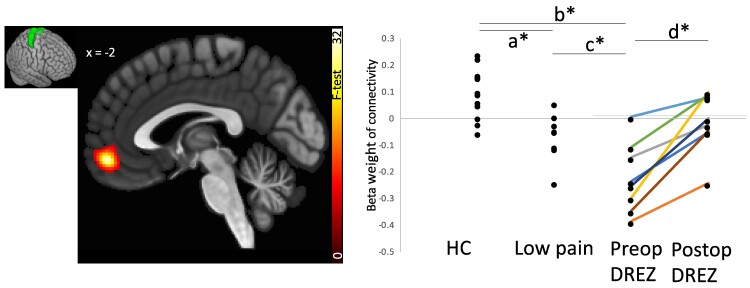
**Group differences and increases in medial post-central seed (illustrated in green in insert) connectivity to the medial prefrontal cortex.** Group ANOVA effects are overlaid on a template MRI, thresholded at *P*_FDR_ < 0.05. Individual connectivity values between the medial prefrontal gyrus and the cluster at MNI_XYZ_ (−2, 52 and −10) were extracted for all groups and plotted for illustrative purposes. *T*-statistics and *P*-values for *post hoc* between and within group tests were (a) *T* = 5.64, *P*_FDR_ = 0.0007, (b) *T* = 6.87, *P*_FDR_ = 0.00006, (c) *T* = 2.22, *P*_FDR_ = 0.044, and (d) *T* = 4, *P*_FDR_ = 0.015. DREZ, dorsal root entry zone; HC, healthy control. * indicates *P*_FDR_ < 0.05.

**Figure 4:**
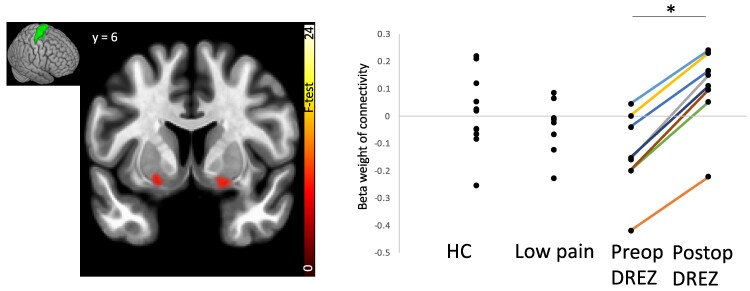
**Increase in rsFC between the medial pre-central gyrus seed (illustrated in green in insert) and the putamen, caudate and accumben region after surgery.** Within-group effects are overlaid on a template MRI, thresholded at *P* < 0.05_FDR_. Individual connectivity values between the medial prefrontal gyrus and the cluster at MNI_XYZ_ (−18, −16 and −14) were extracted for all groups and plotted for illustrative purposes. *T-statistics and *P*-values for *post hoc* within group tests was *T* = 15.83, *P*_FDR_ < 0.000001. DREZ, dorsal root entry zone; HC, healthy controls.

Resting-state connectivity between medial pre- and post-central gyri and the putamen and amygdala were also anticorrelated in pre-DREZ compared with LP and HC. This anticorrelation disappeared after the DREZ surgery and associated pain relief (see Figs 3 and 4 and Supplementary Table 1).

Within the primary somatosensory cortex, Brodmann area 3a displayed a strong anticorrelation to the amygdala hippocampus and accumbens in pre-DREZ compared with LP and HC (*F*(2,25) = 19.8, *P*_FDR_ = 0.0085), which changed from negative to positive after the DREZ intervention (*T*(7) = 11.39, *P*_FDR_ = 0.002). Within the primary motor representation area of the lower body (medial pre-central gyrus), connectivity to the medial frontal cortex and to the bilateral hippocampi was significantly negative in the pre-DREZ group, no connectivity in the LP group and significantly positive in HC (*F*(2,25) = 22.35, *P*_FDR_ = 0.003). After the DREZ intervention, connectivity patterns to the medial prefrontal cortex and hippocampi increased, from anticorrelated to non-correlated (*T*(7) = 3.04, *P*_FDR_ = 0.025).

While the above were the most notable alterations, differences between the groups at baseline and regions that were significantly changed by the intervention for each region of interest are reported and detailed in Supplementary Table 1.

### Functional activation

Finger and foot tapping activated finger and foot primary somatotopic representation areas in S1, M1 and the cerebellum across all groups with overlapping patterns and no significant group or treatment differences (see Supplementary Figs 1A and B).

## Discussion

Substantial below-level neuropathic pain relief was achieved long term in all six patients who underwent DREZ microcoagulation in all regions of DREZ neuronal hyperactivity, i.e. both cephalad and caudal to the level of injury, demonstrating that a neuronally hyperactive DREZ can be causative of below-level SCI neuropathic pain. As in our prior case series of patients with complete spinal transections who achieved pain relief from DREZ microcoagulation only below the spinal lesion, the present study suggests nociceptive transmission via sympathetic afferents, indicative of a novel pain pathway.^3^

Resting-state fMRI revealed group differences between pre-DREZ, LP and HC most evident in lower body primary sensory and motor region connectivity to the medial prefrontal cortex and to the hippocampi and amygdalae. This was largely driven by a strong negative coupling in pre-DREZ as compared with LP and HC. Repeated fMRI performed post-operatively during a time of complete pain relief in all eight patients showed a loss of this negative coupling between the hippocampus and somatosensory cortex and between the amygdala and somatosensory cortex, with post-DREZ subjects becoming similar to LP and HC. In this context, it is also notable that the axonal diffusivity of the uncinate fasciculus, connecting the temporal lobe with the medial orbitofrontal cortex, displayed a decrease post-operatively, consistent with studies contrasting SCI patients with and without pain.^21^ See Supplementary material for details.

Amygdala connectivity to the caudate and to the cerebellum did not differ between groups at baseline but changed from functionally correlated to non-correlated or anticorrelated after the intervention. We speculate that the shift from negative to positive connectivity of motor inter-effector regions to limbic regions may be indicative of motor hypervigilance associated with pain and pain relief, and further studies are needed to define the role of the accumbens in these patterns.

Despite very high levels of neuropathic pain, and despite complete resolution of this pain, we did not observe any significant group differences or within-subject alterations in thalamic or periaqueductal grey connectivity, suggesting that traditional spinothalamic pain pathways were not differentially engaged across groups or altered by the intervention.

Multiple prior studies indicate a role for the hippocampus and amygdala in chronic pain states^22-24^ and a role for primary somatosensory cortex, specifically region BA3a, in C-fibre mediated pain.^25,26^ There are direct projections from deep dorsal horns to the amygdala and orbital cortex.^27^ Moreover, given the prominence of the limbic system in fear and anxiety, it has long been proposed that the transition from acute pain to chronic pain involves a transition from primarily sensory-focused regions to the emotion-centred and limbic areas of the brain.^28^ It has further been proposed that this may progress to the point where a peripheral driver no longer exists, and chronic pain is ‘a persistence of the memory of pain and/or the inability to extinguish the memory of pain evoked by an initial inciting injury’.^29^ This does not seem to be the case in the present study. Instead, our data suggest that hyperactive DREZs are the primary drivers of below-level neuropathic pain and that these engage limbic regions. As such, the concept of a pain memory without peripheral drivers does not apply here. Indeed, along with marked reductions in pain rating, the affective aspects of pain—kinesiophobia, catastrophizing, helplessness and fear—also decreased after the intervention.

Task-induced activations (finger tap and imagined foot tap) did not significantly differ between the pre-op-DREZ patients, LP or HC, nor did the surgery lead to any significant changes in activation patterns. Prior studies indicate reorganization of the sensory cortex in neuropathic pain after SCI,^30^ while our results suggest that classic motor organization is largely unchanged both for the hand and for the feet in persons with SCI both with and without pain and that substantial pain relief after surgery did not alter responsivity in this classic motor organization in the short term.

### Preserved spinothalamic tracts?

Neuropathic pain can be evoked in persons with complete SCI capsaicin sensitization and thermal stimulation below the injury level.^31^ Moreover, up to 50% of persons with clinically complete SCI still exhibit primary somatosensory cortex activation to toe stimulation, so-called discomplete SCI.^32,33^ This has been interpreted as evidence of residual spinothalamic pathways in complete SCI. However, it is well documented that cordectomy does not relieve below-level pain,^34^ indicating that residual spinothalamic pathways are not causal of neuropathic pain. Instead, animal studies suggest that pain develops in parallel with below-level sprouting of C and Aδ primary afferent fibres above and below a lesioned cord.^35^ We suggest that below-level stimuli may sometimes be conducted to the brain via sympathetic afferents.

We find that the present results are consistent with our thesis that deafferentation of the DREZ subsequent to SCI can cause the development of electrically hyperactive second-order pain sensory neurons within the DREZ, both cephalad and caudal to the level of complete SCI, which then become primary generators of below-level pain. This pain perception is dictated by a novel somatotopic map of IML cell column end organ innervation, implicating the involvement of the SNS. Mediation of these electrically hyperactive second-order below-level pain neurons to the brain after SCI may thus be through yet unknown sympathetic afferent pathways ascending the cord directly or through the sympathetic chain, routing around the lesioned cord to engage a limbic–somatosensory–prefrontal circuit, rather than the classical brainstem periaqueductal grey–thalamic–sensory–cortex circuit. We suggest that below-level neuropathic pain is mediated via sympathetic afferents impacting the limbic system and possibly dedicated sympathetic projections to Brodmann area 3a.^25,26^

### Limitations

Two of the study participants elected to only have the DREZ cephalad to the level of injury for fear of losing preserved function had initial pain relief, but a return of pain at 2 months. It is possible that informing that the procedure may not be effective led to some nocebo conditioning in these two participants, but they were pain free at the time of the post-operative MRI. Excluding these two participants from the rsFC analysis lowered the significance of connectivity changes pre- to post-operatively, but the overall patterns remained.

The DREZ surgeries are invasive and can last several hours. Thus, a surgical placebo control, such as equal duration general anaesthesia with only superficial incisions, was not performed.^36^ We note that in our prior study, patients had undergone unsuccessful surgery to their spinal cord prior to benefiting from DREZ.^3^ Moreover, in the present study, participants had tried multiple pharmacological interventions all without sufficient pain relief. Given the highly vulnerable patient population and the invasiveness of the intervention, a randomized trial with placebo surgery would be problematic.

The possibility that below-level pain afferents may re-route across the spinal cord lesion via the sympathetic chain has long been discounted in the literature because of the inability of sympathetic blockade to relieve below-level neuropathic pain.^34^ However, such blockade would need to be both complete and long lasting as well as incorporate all ascending sympathetic cord pathways to rule out a sympathetic driver of below-level pain. Unlike our previous case series with confirmed complete cord transections and DREZ microcoagulation only performed caudal to the level of injury and transection,^3^ we included patients without definitive spinal cord transection, as irrefutable evidence of complete cord transection is rare.

The presence of anticorrelations in brain networks has long been observed.^37,38^ Anticorrelations may be introduced by global signal regression.^39^ Here, we used aCompCor (i.e. the principal component of white matter and CSF signal) as regressors in the pre-processing, but if the method still introduces artificial anticorrelations, this will presumably impact all groups and conditions equally and not drive the current findings. However, it is not clear what such anticorrelation indicates in terms of neuronal processes.

## Conclusions

Our findings in this study have corroborated our previous work,^3,4^ have added new brain connectivity insights and have led us to consider a mechanism of at-level and below-level SCI neuropathic pain in the paraplegic population. We propose that neuropathic pain develops when SCI leads to deafferentation of the DREZ, particularly C-fibre deafferentation and/or loss of descending DREZ inhibition, neuronal hyperexcitability of second-order neurons ensuing in the DREZ in regions inclusive of Rexed laminae 1–3. If DREZ hyperactivity becomes mediated by classical pain pathways, e.g. spinothalamic tract to thalamus to sensory cortex, then the pain will be perceived at level. If, however, the hyperactivity becomes mediated wholly or in part through the SNS, by way of a newly created neuronal circuit possibly incorporating the IML cell column, sympathetic chain and ascending SNS afferent cord pathways, we propose that pain will be perceived below level corresponding to body regions of IML cell column end organ innervation, along with the manifestation of negative rsFC of the somatomotor cortex to the hippocampus amygdala and medial prefrontal cortex. Our findings are consistent with the duality of a DREZ in mediating neuropathic pain, utilizing both classical, at-level, and non-classical, below-level, SNS pain pathways.

An intriguing possibility is that the pain system is composed of both spinothalamic and sympathetic second-order afferent projections and that these two parallel sources are normally compared. We speculate that after SCI and development of DREZ hyperactivity, the consequent classical spinal and sympathetic signaling mismatch engages an ‘alarm system’ of anticorrelation between somatosensory to limbic and medial prefrontal—and motor to caudate—connectivity. This suggests a previously overlooked pain transmission mechanism that may be active also in other neuropathic pain syndromes.

## Supplementary Material

fcae411_Supplementary_Data

## Data Availability

Anonymized data that support the findings of this study are available on request from the corresponding author. The data are not publicly available due to their containing information that could compromise the privacy of research participants.
