# Supplementary material for: Neuropathic pain relief and altered brain networks after dorsal root entry zone microcoagulation in patients with spinal cord injury
Source: Brain Commun. 2024 Nov 21;6(6):fcae411. doi: 10.1093/braincomms/fcae411 (PMC11601164; doi:10.1093/braincomms/fcae411)
Supplement: fcae411_Supplementary_Data [file fcae411_supplementary_data.pdf]

Supplemental materials

Supplemental methods

Data acquisition parameters

T1 weighted structural images were acquired with a gradient echo sequence with GRAPPA parallel imaging with an acceleration factor of 2, 256 mm field of view (FOV), 1x1x1 mm voxel size, 1 mm slice thickness, sagittal acquisition (interleaved), 20 ms echo time (TE), 4.92 ms TR, flip angle of 25 degrees, and 5:17 scan time.

An echo-planar imaging sequence with a 216 mm FOV, 3x3x3 mm voxel size, 3 mm slice thickness, acquisition interleaved, 30 ms TE, 3000 ms TR, flip angle of 85 degrees and 6:18 scan time (2 repeated scans collected in each subject) was used to acquire resting state functional images in all subjects.

A finger-tap and a foot-tap experimental paradigm was collected in all subjects. The EPI sequence had a 200 mm FOV, 3.1x3.1x3.1 mm voxel size, interleaved acquisition, 30 ms TE, 3000 ms TR, flip angle of 90 degrees, and a 4:06 scan time. One finger tap and one foot tap paradigm was collected, with a blocked design of four sets of 30 second blocks of tapping, interleaved with rest. Participants unable to tap their feet due to paralysis were asked to attempt and imagine tapping.

Diffusion-weighted imaging (DWI) was acquired at each study time point for white-matter analyses. The DWI scanning sequence included 46 directions with two diffusion weights: 0 s/mm^2 and 1000 s/mm^2, and the slices were collected in the A-P one phase-encoding direction. DWI data were acquired using the GRAPPA parallel imaging technique (TR 7100 ms, TE 88 ms, FOV 350 mm, slice thickness 2.7 mm, 59 contiguous transversal slices, acceleration factor 2, voxel size 2.7 x 2.7 x 2.7 mm, Epi factor 84, with a total scan time of 5 minutes and 55 seconds.

Follow up analyses of motor and somatosensory regions

Recent evidence indicates that the classic motor homunculus is in punctuated by a system for whole-body action planning with distinct connectivity profiles, so called inter-effector regions.[1] Moreover, the primary somatosensory cortex can be subdivided cytoarchitectonically into four distinct Brodmann areas (BA) (1, 2, 3a and 3b).[2] In light of this, we further explored rsFC alterations in the motor-effector regions, provided by Gordon *et al.* [1], which were grouped into

three bilateral groups (superior (i.e. “between” lower and upper limbs), middle (i.e. between upper limbs and face) and inferior (below face), and rsFC alteration in probabilistic delineations of somatosensory BA 1, 2, 3a and 3b.[2]

Cortical thickness

T1 brain images were analyzed using *FreeSurfer*.[3-7] Cortical clusters were smoothed at 10mm full-width at half-maximum (FWHM). A vertex wise threshold of $p < 0.001$ was used with a one-sided p-threshold < 0.05. Group differences were evaluated using ANOVA. For pre- to post-operative changes in cortical thickness, we utilized the longitudinal stream in *FreeSurfer*.[7]

Voxel-wise diffusion tensor imaging and tract analysis

Diffusion-weighted imaging was analyzed using a combination of, MRtrix3,[8] FSL 6.0.4 (FMRIB's Software Library),[9] TRActs Constrained by UnderLying Anatomy (TRACULA),[10] and Pointwise Assessment of Streamline Tractography Attributes (PASTA) [11] to determine fractional anisotropy (FA), mean diffusivity (MD), radial diffusivity (RD), and axial diffusivity (AD) values along the trajectory of each participant's tracts, then performed group comparisons and pre- and post-DREZ of the microstructural integrity.

The diffusion-weighted imaging (DWI) analysis pipeline employed the MRtrix3 tool[8] and FSL 6.0.4 (FMRIB's Software Library) [9,12,13] for data pre-processing, which involved denoising with Marchenko–Pastur principal component analysis [14,15] to remove noise while preserving sharp features and correction for Gibbs artifacts [16] to reduce image distortions. Eddy-FSL was used for the correction of eddy current-induced and motion-induced distortion [17,18]. Eddyqc tool from FSL was used to perform quality assessment on preprocessed dMRI, and study-wise quality assessment for DWI (SQUAD) was used to exclude outliers [19]. The images were skull-stripped, and masks were created using the Brain Extraction Tool (BET) [20]. The tensor model was then applied to the adjusted DWI data, creating fractional anisotropy (FA) derived from eigenvalues and eigenvectors using DTIFIT [13].

We assessed the white matter microstructure using Tract-Based Spatial Statistics (TBSS) [21] available in FSL version 6.0.6, to conduct a voxel-wise examination of FA maps. We removed brain-edge artifacts using TBSS preprocessing and performed nonlinear registration and alignment of all the FA data across subjects using TBSS registration. We merged all subjects'

standard spaces nonlinearly aligned images to create an FA skeleton and performed standard space registration in a 1x1x1mm MNI152 image using TBSS post-registration [21]. For the FA skeleton generation, we used a threshold of 0.4 to exclude voxels whose origin was cerebrospinal fluid or gray matter and projected the pre-aligned FA data onto the mean FA skeleton using TBSS pre-stats. We employed the nonparametric permutation-based analysis available in FSL (FSL's randomize function) [22] to perform the voxelwise statistics of the FA differences between the baseline and post-DREZ groups. We utilized the Threshold-Free Cluster Enhancement (TFCE) method at a significance level of $p < 0.05$ with 5,000 permutations fully adjusted for multiple comparisons to identify enhanced signal clusters within the FA data when comparing the groups pre-post DREZ.

We assessed the white-matter integrity using TRACULA [10,23] (TRActs Constrained by UnderLying Anatomy), a tool within the FreeSurfer software suite. We utilized Freesurfer version 7.4.0 for each participant and its recon-all function to perform automated motion correction, Tailarach transformation, cortical parcellation, and volumetric and subcortical segmentation of T1-weighted images. We performed intra-subject registration (from each subject's DWI to the subjects' T1-weighted image) using bbregister, a Freesurfer tool that optimize the affine registration using the subjects' surface reconstruction and performed inter-subject registration using affine registration from the individual T1 to the MNI T1 template using FLIRT (FMRIB's Linear Image Registration Tool from FSL) [24]. We then applied TRACULA's standard tensor fitting and tract reconstruction processes, using the ball-and-stick model (BEDPOSTX) [25-28], to the prepared data for estimating diffusion probabilities (FMRIB Software Library, Oxford). Following this, we used TRACULA to generate the probability distributions of forty-two white matter tracts [10,23]. We performed a quality assessment with TRACULA's recommended pipeline assessing average volume-by-volume translation, average volume-by-volume rotation, percent of slices with excessive intensity drop-out, and the average drop-out score for slices with excessive intensity drop-out.

We performed along-tract statistical analysis using the Pointwise Assessment of Streamline Tractography Attributes (PASTA) [11], which involved the extraction of fractional anisotropy (FA), mean diffusivity (MD), radial diffusivity (RD), and axial diffusivity (AD) values along the trajectory of each participant's tracts, then performed group comparisons and pre- and post-DREZ of the microstructural integrity. To assess the variation of these diffusivity measures, we

fitted a general linear model (GLM) at each point along the tracts. We used mri_glmfit for the initial model fitting and mri_glmfit-sim for permutation-based cluster-wise correction (Freesurfer 7.4.0) at a significance level of $p < 0.05$ with 5,000 permutations.

Supplementary results

Seed based resting state functional connectivity.

Medial precentral gyrus

The main finding of the between group ANOVA analysis was a difference in resting state connectivity between the primary motor representation area of the lower body (medial precentral gyrus) to the medial frontal cortex. Post-hoc analyses indicated that this was driven by negative connectivity in the preop-DREZ group, a more modest anticorrelation in LP, and a significant positive connectivity in HC. Within the DREZ group, there was a significant increase in connectivity to the medial frontal cortex after the DREZ intervention, in that patterns changed from negative to weak positive correlation.

The between group ANOVA further revealed a significant difference in medial precentral connectivity to the left and right hippocampus. Post-hoc analysis indicated that the preop-DREZ connectivity was significantly lower than in both LP and HC. Examining the direction of connectivity, preop-DREZ subjects again displayed modest negative connectivity between the medial postcentral gyrus and the bilateral hippocampi, whereas the HC group displayed significant positive connectivity in these nodes. The LP fell in between, with no significant correlation or anticorrelations. This connectivity pattern was not significantly changed by the intervention, however, directly examining treatment induced connectivity differences of the medial postcentral gyrus within the DREZ group revealed significant increases (from negative connectivity to positive connectivity) to the bilateral putamen/amygdala and to the anterior cingulate, medial frontal cortex and left thalamus.

Medial postcentral gyrus

ANOVA analysis indicated group differences in rsFC to the medial frontal cortex and the bilateral hippocampi. Post-hoc analysis indicated that connectivity alterations to the medial frontal cortex were driven by negative connectivity in both the preop-DREZ and the LP group as compared to a significant positive connectivity in HC. For the connectivity to the hippocampi, post-hoc analysis revealed a significantly lower connectivity in the preop-DREZ (modest negative connectivity), with no connectivity in the LP and a significant positive connectivity in HC.

The DREZ intervention led to a significant increase in medial postcentral connectivity to the anterior and posterior cingulate in that connectivity patterns shifted from moderately anticorrelated to zero-correlation.

Right Anterior Insula

Between the three groups, there was a significant difference in right anterior insula connectivity to two clusters in the left cerebellum; cerebellar region 6 and crus 1 and cerebellar region 8 and 9. Post-hoc analysis revealed that for cerebellar region 6 and crus 1, this was driven by a significant correlation in HC that was lower in both the preop-DREZ and in LP. For cerebellar region 8 and 9, rsFC was anticorrelated in the preop-DREZ, low in LP, and significantly correlated in the HC group.

The DREZ intervention did not significantly alter rsFC of the right anterior insula.

Left Anterior Insula

No significant group or treatment changes observed.

Right posterior insula

No significant group or treatment changes observed.

Left Posterior Insula

Between the three groups, there was a significant difference in connectivity to clusters in the precuneus cortex, the right frontal pole and bilateral cerebellum.

Post-hoc analysis revealed that for the precuneus cluster, rsFC was significantly lower in the preop-DREZ group than in LP. Notably, the preop-DREZ did not differ from HC, rather, the LP

group displayed significant rsFC, whereas preop-DREZ and HC groups displayed no significant connectivity. The DREZ intervention did not significantly alter connectivity to this cluster.

For the right frontal pole, preop-DREZ and HC displayed higher connectivity than LP. This effect was driven by a negative connectivity in LP, a positive connectivity in the HC group, and a non-significant positive connectivity in the preop-DREZ. The DREZ intervention did not significantly change connectivity of the posterior insula.

The DREZ intervention led to a significant increase in connectivity (from negative to positive) to the right amygdala.

Anterior Cingulate

Between the three groups, there were significant difference in connectivity to the right precentral gyrus, and to the right superior frontal gyrus. Post-hoc analysis revealed that connectivity to the right precentral gyrus was significant in the HC group, but lower and non-significant in both SCI groups. A different pattern was evident for the right superior frontal gyrus, where the LP group had greater connectivity than both the preop-DREZ and the HC group. The DREZ intervention did not significantly alter connectivity to these clusters.

The DREZ intervention led to an increase from modest anticorrelation to significant correlation to the left precentral gyrus.

Posterior mid-cingulate cortex (PMCC)

Between the three groups, there were no significant difference in posterior mid-cingulate connectivity.

The DREZ intervention led to a decrease, from a very high rsFC to a more moderate but still significant rsFC to the middle frontal gyrus. Notably, the LP and HC groups also displayed this significant rsFC between PMCC and the middle frontal gyrus.

A significant decrease post-intervention in PMCC connectivity to the right occipital pole was also observed.

Amygdala

Between the three groups there were no significant difference in right or left amygdala connectivity.

The DREZ intervention led to multiple changes:
A marked decrease in right amygdala connectivity to the caudate, and in left amygdala connectivity to the cerebellar vermis.
Consistent with the analyses above using the medial pre- and post-central gyri as seeds, there was a significant increase in amygdala connectivity to pre- and post-central gyrus, with connectivity typically changing from moderately anticorrelated to moderately correlated. In addition to changes in rsFC to primary somatomotor regions, the right amygdala displayed a significant increase in connectivity to the pre-motor cortex (Superior frontal gyrus), from anti-correlated to correlated rsFC, and increased connectivity to the planum temporale.

Hippocampus
Between the three groups, hippocampal connectivity differed to the medial pre- and post-central gyrus, as was observed using the medial pre- and post-central gyri as seeds. Post-hoc analysis revealed that the preop-DREZ group was significantly more negatively correlated than LP and HC, in that preop-DREZ subjects displayed significant anti-correlation, LP no correlation, and HC significant correlation.
The DREZ intervention led to changes in hippocampal connectivity the cerebellum, in that connectivity between hippocampus and the cerebellum decreased. Moreover, right hippocampus connectivity to the right superior frontal gyrus increased significantly, from negative to non-correlated. Of note, changes in connectivity to the medial pre- and post-central gyrus were not significant in this contrast.

Thalamus and periaqueductal gray
There were no significant effects of group, or of the DREZ intervention.

Follow up analyses of motor and somatosensory regions
In our follow-up analysis of the motor cortex, we evaluated changes in rsFC in three proposed motor inter-effector regions [1]. Group differences in rsFC between DREZ, low pain SCI and HC were evident in the superior motor effectors connectivity to the hippocampi, caudate and medial prefrontal regions. For the hippocampi and medial prefrontal regions, these differences were driven by a negative connectivity in both the pre-op DREZ and in LP as compared to HC. For the

caudate, the difference was driven by a negative connectivity in the DREZ group versus a positive connectivity in the HC, with LP displaying a moderate negative connectivity.

Contrasting pre-operative to post-operative rsFC in the DREZ group indicated a significant change, from moderately negative to moderately positive rsFC to the cerebellum (crus 1 and 6) and to the thalamus. The LP and HC displayed no significant rsFC to these regions.

The middle inter-effector region displayed a significant group difference in connectivity to a large cluster in the accumbens region. This effect was uniquely driven by a negative connectivity in the preop-DREZ group, and the middle inter-effector to accumbens connectivity further displayed a significant increase in rsFC post-operatively.

Contrasting pre-operative to post-operative rsFC in the DREZ group indicated no significant changes at our threshold of $p_{FDR}<0.0036$, while results at a less stringent threshold ($p_{FDR}<0.05$) were consistent with rsFC connectivity changes to the medial precentral gyrus reported above.

No group differences were observed in connectivity of the inferior inter-effector regions, nor were any treatment effects observed.

Within the primary somatosensory cortex, BA 1 displayed a group difference in connectivity to the medial frontal cortex, and, notably, this was driven by a significant positive connectivity in the LP group, whereas the preop-DREZ group displayed negative coupling and HC no significant connectivity. The BA 2 subdivision of the somatosensory cortex displayed no group or treatment changes.

BA 3a and 3b both displayed anticorrelations in the preop-DREZ group as compared to both LP and HC. BA 3a further displayed anticorrelations to the to the accumbens, as compared to both LP and HC. A group difference in connectivity to the medial frontal cortex was also observed, driven by a negative correlation in the preop-DREZ group and a positive correlation in the HC, with LP falling in between.

After the DREZ intervention, rsFC of BA 3a and 3b to the right amygdala changed from anticorrelated to correlated. This effect was observed also for connectivity between BA 3a and the left amygdala.

Structural differences

There were no significant group differences or changes pre- to post-operative in cortical thickness.

Voxel-wise diffusion tensor imaging (DTI) and tract analysis differences across groups

A comparison between the preoperative DREZ and LP groups using TRACULA and PASTA analysis indicated differences in several white matter tracts. For axial diffusivity (AD), we observed a cluster in the left anterior thalamic radiation consisting of 12 voxels with a volume of 12.0 mm^3 (T = -2.55 and a cluster-wise p-value (p_{CW}) of 0.0002). Similarly, the left cingulum bundle exhibited a cluster of 26 voxels, measuring 26.0 mm^3 (T= -3.08, p_{CW}= 0.0002). The right acoustic radiation also showed a significant cluster (15.0 mm^3, T= -2.64, p_{CW}= 0.0002). In mean diffusivity (MD) analysis, the right acoustic radiation demonstrated a cluster (17.0 mm^3, T= -3.37, p_{CW}= 0.0002). The right frontal aslant tract displayed a cluster (15.0 mm^3, T= -3.02, p_{CW}= 0.029). Additionally, the right superior longitudinal fasciculus (temporal part) revealed a significant cluster (26.0 mm^3, T= -3.19, p_{CW}= 0.0002). For radial diffusivity (RD), significant clusters were detected in the corpus callosum's body (27.0 mm^3, T= -2.26, p_{CW}= 0.025); the corpus callosum's rostrum (9.0 mm^3, T= -2.33, p_{CW}= 0.026), and the left corticospinal tract (22.0 mm^3, (T= -2.69, p_{CW}= 0.0002). For fractional anisotropy (FA), no significant differences were observed. Microstructural results using TBSS revealed no significant clusters in the TFCE of the TBSS when comparing the FA in preoperative DREZ and LP groups.

Changes in Voxel-wise diffusion tensor imaging (DTI) and tract analysis after DREZ

Within the DREZ group, our results using TRACULA and PASTA revealed no significant alterations in FA, MD, or RD when comparing the pre- and postoperative DREZ surgery groups. In AD analysis, we identified a cluster (19.0 mm^3, T= -1.94, p_{CW}= 0.036, lower and upper bounds of 0.033 and 0.04) within the left uncinate fasciculus when comparing the pre- and postoperative DREZ surgery groups. Microstructural results using TBSS revealed no significant clusters in the TFCE of the TBSS when comparing the FA in pre- and post-DREZ groups.

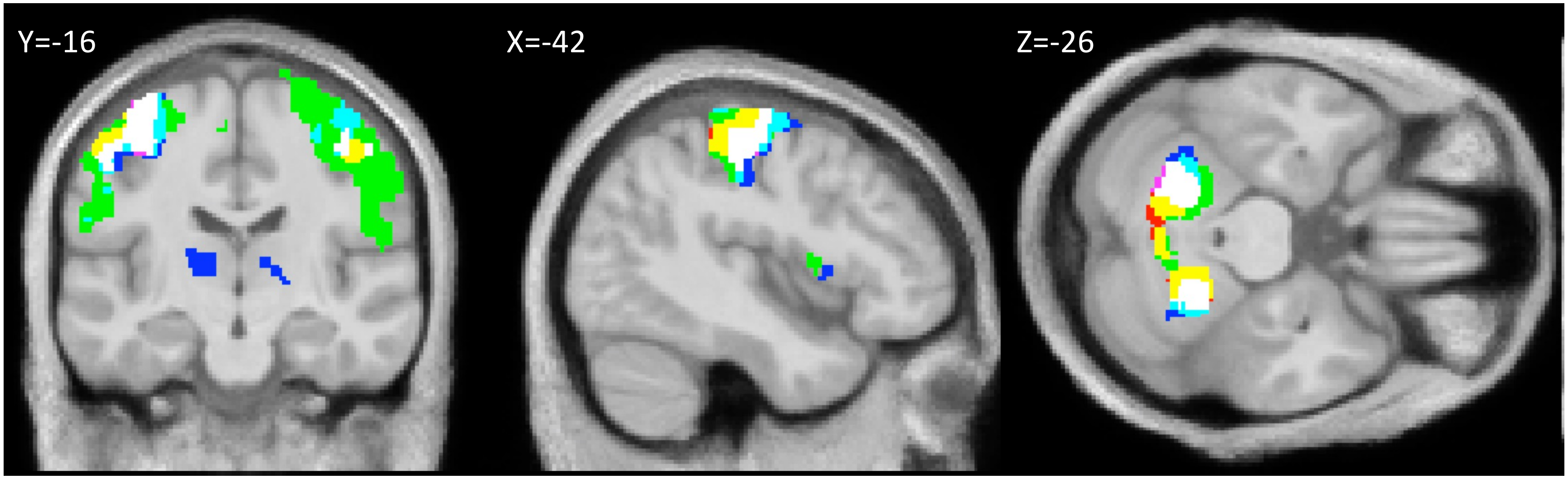

Supplemental Figure 1a. Finger tapping led to significant activation of the precentral and post-central gyrus in the expected region of finger representation, and of the bilateral cerebellum. Image indicates activated regions thresholded at T>3.5 (p<0.001), where green represents healthy controls, blue represents low pain SCI subjects, and red represents DREZ SCI subjects with pain prior to DREZ intervention. There were no significant differences between the three groups, and no significant changes in the finger-tap activation pattern after the DREZ surgery.

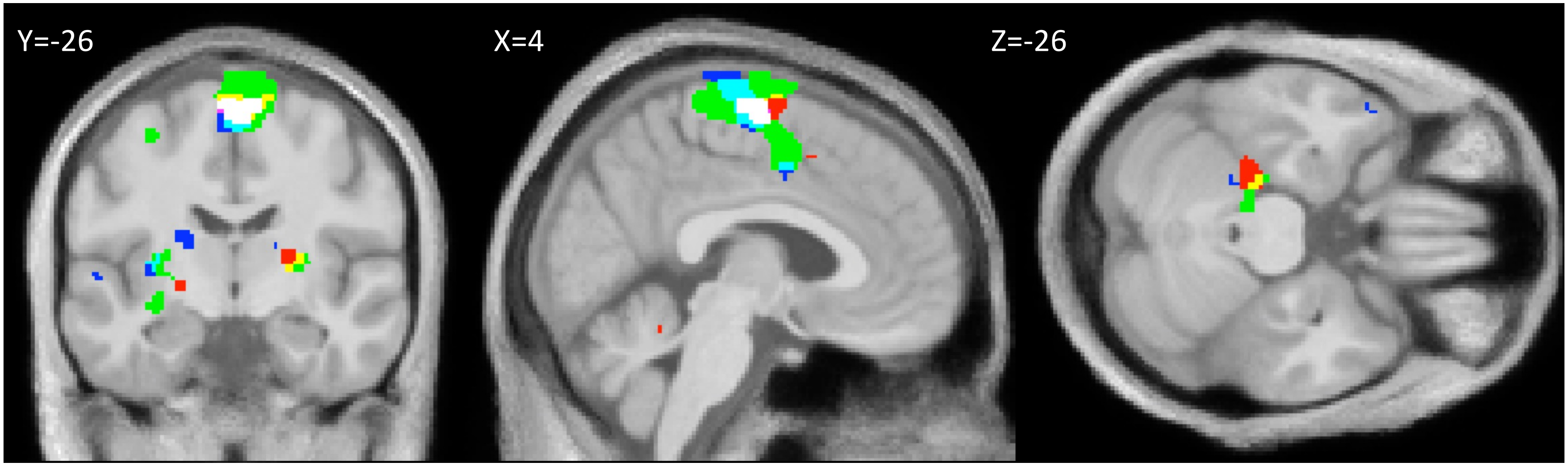

Supplemental figure 1b. Foot tapping (or imagining foot-tapping) led to significant activation of the precentral and post-central gyrus in the expected region of foot representation, and of the bilateral cerebellum. Image indicates activated regions thresholded at T>3.5 (p<0.001), where green represents healthy controls, blue represents low pain SCI subjects, and red represents DREZ SCI subjects with pain prior to DREZ intervention. Complete overlap is indicated in white. There were no significant differences between the three groups, and no significant changes after the DREZ surgery.

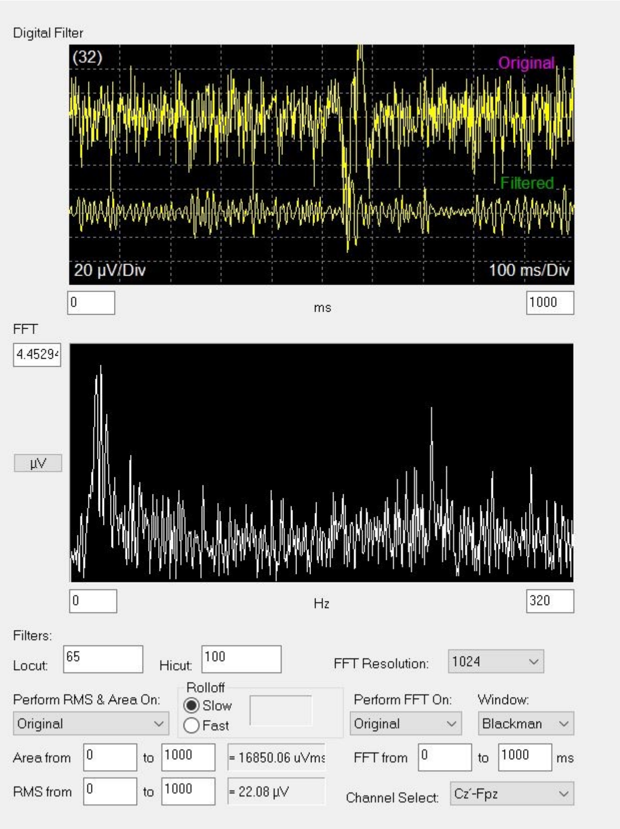

Hyperactive DREZ

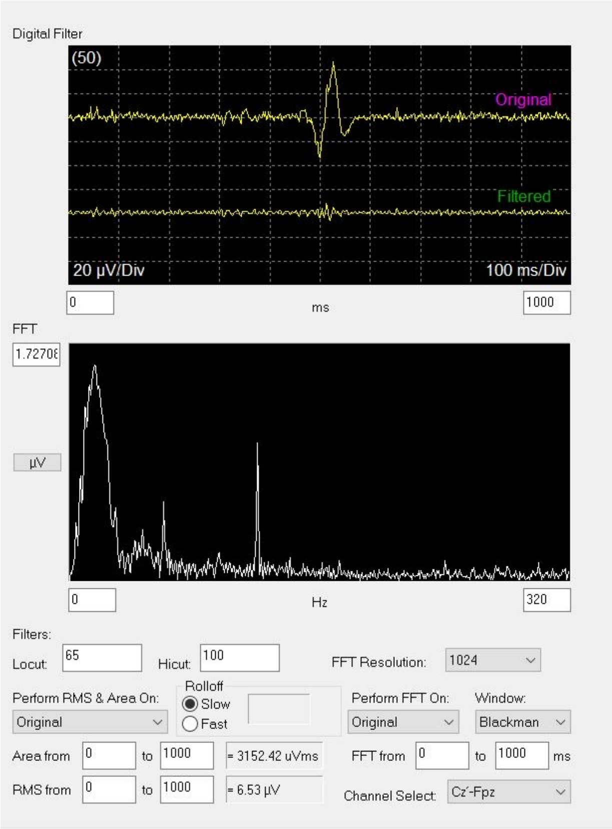

DREZ after lesioning

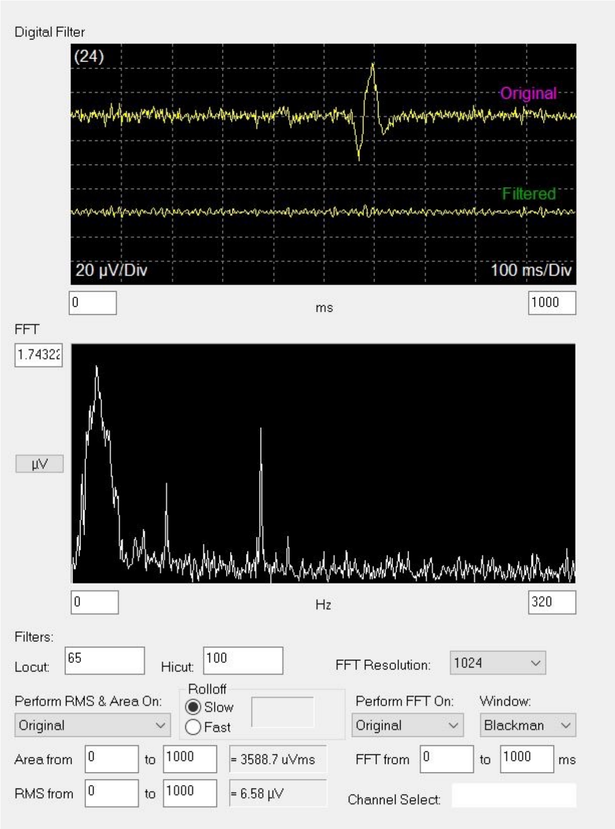

”Cold” DREZ (not lesioned)

Supplemental figure 2. Examples of dorsal root entry zone recordings in a single patient. Top panels (yellow) indicates measured activity. Bottom panel indicates bandpass filtered activations (65-100hz) that are Fast Fourier Transferred to identify spindels.

Seed	Contrast	Resulting region	MNI x	MNI y	MNI z	cluster size	P_{FDR}	Posthoc contrast	T	P_{FDR}	Connectivity	T	P_{FDR}
**Medial precentral**	**Group ANOVA**	Medial frontal cortex	-2	50	-14	737	<0.000001*	preop-DREZ < low-pain SCI	0.92	0.37			
								preop-DREZ < HC	7.42	0.000001*			
								low-pain SCI<HC	6.98	0.00002*			
								Preop < Postop	4.77	0.002*			
											pre-DREZ	-2.8	0.035
											post-DREZ	1.11	0.59
											low-pain SCI	-1.93	0.38
											HC	8.77	0.000011*
		R. hippocampus and amygdala	22	-12	-16	333	0.00031*	preop-DREZ < low-pain SCI	2.38	0.064			
								preop-DREZ < HC	7.1	0.000001*			
								low-pain SCI<HC	3.85	0.00012*			
								Preop < Postop	3.04	0.025			
											pre-DREZ	-3.59	0.035
											post-DREZ	0.81	0.59
											low-pain SCI	-0.12	0.91
											HC	6.95	0.000032*
		L. hippocampus	-36	-24	-14	217	0.00025*	preop-DREZ < low-pain SCI	2.66	0.064			
								preop-DREZ < HC	6.51	0.000004*			
								low-pain SCI<HC	3.95	0.00093*			
								Preop < Postop	3.2	0.025			
											pre-DREZ	-2.98	0.035
											post-DREZ	0.81	0.59
											low-pain SCI	0.38	0.91
											HC	7.52	0.00023*
		R. posterior supramarginal gyrus	52	-36	34	113	0.045	preop-DREZ < low-pain SCI	1.02	0.37			
								preop-DREZ < HC	4.13	0.00063*			
								low-pain SCI<HC	3.95	0.00093*			
								Preop < Postop	1.08	0.31			
											pre-DREZ	1.28	0.24
											post-DREZ	0.43	0.68
											low-pain SCI	0.44	0.91
											HC	-4.9	0.00047*
	**Pre < post DREZ**	L. Putamen and Amygdala	-18	-16	-14	282	<0.000001*				pre-DREZ	-2.7	0.031
											post-DREZ	2.02	0.17
											low-pain SCI	-1.04	0.5
											HC	0.26	0.92
		R. Putamen and Amygdala	18	6	-10	216	<0.000001*				pre-DREZ	-3.74	0.011
											post-DREZ	3.2	0.045
											low-pain SCI	-0.45	0.67
											HC	0.1	0.92
		Anterior Cingulate	10	40	8	129	0.00026*				pre-DREZ	-4.57	0.0069
											post-DREZ	1.22	0.31
											low-pain SCI	-0.77	0.56
											HC	-0.24	0.92
		Medial Frontal Cortex	6	48	-14	57	0.022				pre-DREZ	-3.24	0.017
											post-DREZ	1.72	0.19

											low-pain SCI	-1.21	0.5
											HC	3.32	0.021
		L. Thalamus	-2	-4	8	46	0.035				pre-DREZ	-4.33	0.0069
											post-DREZ	1.09	0.31
											low-pain SCI	-1.97	0.27
											HC	-0.51	0.92
	**Pre > post DREZ**	R. Precentral Gyrus	50	-2	24	51	0.028				pre-DREZ	7.16	0.0002*
											post-DREZ	3.61	0.045
											low-pain SCI	3.96	0.033
											HC	5.39	0.0013*
**Medial postcentral**	**Group ANOVA**	Medial Frontal Cortex	-2	52	-10	650	<0.000001*	preop-DREZ < low-pain SCI	2.22	0.044			
								preop-DREZ < HC	6.87	0.00006*			
								low-pain SCI<HC	5.64	0.00071*			
								Preop < Postop	4	0.015			
											pre-DREZ	-4.63	0.0036
											post-DREZ	-0.37	0.87
											low-pain SCI	-3.52	0.029
											HC	4.77	0.0011*
		L. Hippocampus	-22	-22	-18	225	0.0029*	preop-DREZ < low-pain SCI	2.41	0.044			
								preop-DREZ < HC	5.55	0.000029*			
								low-pain SCI<HC	3.76	0.0021			
								Preop < Postop	2.3	0.055			
											pre-DREZ	-3.28	0.013
											post-DREZ	-0.47	0.87
											low-pain SCI	-0.84	0.43
											HC	4.65	0.0011*
		R. Hippocampus	20	-26	-14	159	0.012	preop-DREZ < low-pain SCI	2.64	0.044			
								preop-DREZ < HC	5.79	0.00026*			
								low-pain SCI<HC	3.11	0.006			
								Preop < Postop	2.57	0.055			
											pre-DREZ	-4.64	0.0036
											post-DREZ	-0.17	0.87
											low-pain SCI	-1.28	0.36
											HC	3.33	0.0067*
	**Pre < post DREZ**	Anterior Cingulate	8	36	12	420	<0.00001*				pre-DREZ	-4.89	0.0036
											post-DREZ	-0.27	0.79
											low-pain SCI	-2.05	0.16
											HC	-0.04	0.96
		Posterior Cingulate	0	-18	36	60	0.035				pre-DREZ	-2.55	0.038
											post-DREZ	0.69	0.79
											low-pain SCI	-1.4	0.21
											HC	-1.24	0.48
**Left anterior insula**	**Group ANOVA**	none											
	**Pre-post DREZ**	none											
**Right anterior insula**	**Group ANOVA**	L. cerebellar region 6 and crus 1	-44	-54	-36	286	0.00046*	preop-DREZ < low-pain SCI	1.62	0.13			
								preop-DREZ < HC	6.76	0.0000021*			
								low-pain SCI<HC	4.75	0.0003*			
								Preop < Postop	0.45	0.7			

											pre-DREZ	-0.15	0.89
											post-DREZ	0.45	0.79
											low-pain SCI	1.93	0.19
											HC	9.42	0.000003*
		L. cerebellar region 8 and 9	-28	-40	-44	142	0.015	preop-DREZ < low-pain SCI	3.35	0.009			
								preop-DREZ < HC	9.03	0.00001*			
								low-pain SCI<HC	4.31	0.0004*			
								Preop < Postop	2.39	0.097			
											pre-DREZ	-4.93	0.0034*
											post-DREZ	0.27	0.79
											low-pain SCI	0.2	0.84
											HC	8.19	0.000005*
	**Pre-post DREZ**	none											
**Left posterior insula**	**Group ANOVA**	precuenous cortex	-18	-60	20	175	0.004*	preop-DREZ < low-pain SCI	5.93	0.00006*			
								preop-DREZ < HC	0.08	0.93			
								low-pain SCI<HC	6.64	0.00005*			
								Preop < Postop	0.64	0.54			
											pre-DREZ	-0.32	0.76
											post-DREZ	0.6	0.88
											low-pain SCI	8.16	0.00013
											HC	-0.29	0.78
		R. frontal pole	4	56	-28	163	0.004*	DREZ > Pain free	6.02	0.000061*			
								preop-DREZ < HC	1.18	0.31			
								low-pain SCI<HC	9.08	<0.000001*			
								Preop > Postop	1.49	0.28			
											pre-DREZ	2.11	0.18
											post-DREZ	-0.16	0.88
											low-pain SCI	-8.78	0.00013*
											HC	5.4	0.0011*
		L. frontal pole	-14	48	-16	85	0.042	DREZ > Pain free	2.86	0.018			
								preop-DREZ < HC	4.46	0.0015*			
								low-pain SCI<HC	6.84	0.000005*			
								Preop < Postop	1.37	0.28			
											pre-DREZ	-2.18	0.18
											post-DREZ	0.24	0.88
											low-pain SCI	-4.92	0.0017*
											HC	4.37	0.0028*
		R. cerebellum area 4 and 5	12	-42	-22	126	0.011	preop-DREZ < low-pain SCI	5.73	0.000065*			
								preop-DREZ < HC	2.35	0.067			
								low-pain SCI<HC	4.66	0.00019*			
								Preop < Postop	1.81	0.28			
											pre-DREZ	-1.65	0.24
											post-DREZ	0.94	0.88
											low-pain SCI	7.23	0.00022*
											HC	1.57	0.18
		L. Cerebellar area 4 and 5	-18	-42	-18	70	0.043	preop-DREZ < low-pain SCI	7.09	0.000027*			
								preop-DREZ < HC	2.21	0.067			
								low-pain SCI<HC	5.29	0.000062*			

								Preop < Postop	1.33	0.28			
											pre-DREZ	-0.81	0.56
											post-DREZ	1.07	0.88
											low-pain SCI	11.64	0.000039*
											HC	2.53	0.047
	**Preop < post DREZ**	R. amygdala	24	-2	-16	50	0.048				pre-DREZ	-2.72	0.03
											post-DREZ	6.26	0.0004*
											low-pain SCI	2.78	0.027
											HC	1.4	0.18
**right posterior insula**	**Group ANOVA**	none											
	**Pre-post DREZ**	none											
**Anterior cingulate**	**Group ANOVA**	R. precentral gyrus	16	-26	36	116	<0.00001*	DREZ < Pain free	0.19	0.85			
								preop-DREZ < HC	6.29	0.000012*			
								low-pain SCI<HC	6.09	0.000019*			
								Preop < Postop	1.44	0.38			
											pre-DREZ	1.84	0.21
											post-DREZ	2.44	0.09
											low-pain SCI	2.02	0.084
											HC	9.97	0.00002*
		R. superior frontal gyrus	28	14	58	110	0.000018*	preop-DREZ < low-pain SCI	4.09	0.002*			
								preop-DREZ < HC	1.1	0.29			
								low-pain SCI<HC	5.09	0.000077*			
								Preop < Postop	0.77	0.47			
											pre-DREZ	-0.17	0.87
											post-DREZ	-0.89	0.4
											low-pain SCI	4.53	0.0054
											HC	-1.72	0.11
	**Pre < post DREZ**	L. postcentral gyrus	-34	-34	64	69	0.000039*				pre-DREZ	-0.94	0.38
											post-DREZ	3.49	0.001*
											low-pain SCI	-1.25	0.25
											HC	-0.08	0.94
**Posterior cingulate**	**Group ANOVA**	none											
	**Pre>post DREZ**	R. occipital pole	20	-100	-2	105	<0.000001*				pre-DREZ	1.07	0.32
											post-DREZ	-3.26	0.014
											low-pain SCI	-0.7	0.5
											HC	1.76	0.11
		L. middle forntal gyrus	-32	18	48	50	0.000025*				pre-DREZ	10.11	0.00004*
											post-DREZ	5.21	0.0025*
											low-pain SCI	4.37	0.0066
											HC	5.59	0.00033*
**Left Amygdala**	**Group ANOVA**	none											
	**Pre>post DREZ**	Cerebellar vermis	4	-72	-28	221	0.000017*				pre-DREZ	3.31	0.052
											post-DREZ	-3.43	0.022
											low-pain SCI	0.9	0.79
											HC	1.14	0.36
		L. planum temporale	-54	-30	12	52	0.000012*				pre-DREZ	-1.17	0.28
											post-DREZ	6.92	0.0009*
											low-pain SCI	1.22	0.79
											HC	4.09	0.0072*

		R. precentral gyrus	20	-16	76	42	0.000011*				pre-DREZ	-1.91	0.19
											post-DREZ	2.28	0.076
											low-pain SCI	-0.22	0.83
											HC	3.13	0.019
		R. precentral gyrus	32	-16	70	41	0.00076*				pre-DREZ	-1.3	0.28
											post-DREZ	1.48	0.18
											low-pain SCI	0.39	0.83
											HC	0.96	0.36
**Right Amygdala**	**Group ANOVA**	none											
	**Pre>post DREZ**	R. caudate	14	20	8	199	0.000001*				pre-DREZ	8.8	0.00039*
											post-DREZ	-0.85	0.42
											low-pain SCI	2.97	0.14
											HC	3.23	0.032
		R. precentral gyrus	12	-32	66	106	0.000009*				pre-DREZ	-2.84	0.031
											post-DREZ	1.46	0.21
											low-pain SCI	-0.12	0.97
											HC	2.63	0.063
		L. planum temporale	-40	-32	14	93	0.000024*				pre-DREZ	-1.57	0.16
											post-DREZ	4.27	0.01
											low-pain SCI	2.59	0.14
											HC	2.17	0.084
		superior frontal gyrus	26	-10	68	68	0.000022*				pre-DREZ	-3.03	0.032
											post-DREZ	4.36	0.01
											low-pain SCI	-0.03	0.97
											HC	1.06	0.31
		L. supplementary motor cortex	-10	-8	58	65	0.000016*				pre-DREZ	-2.92	0.032
											post-DREZ	3.83	0.013
											low-pain SCI	1.66	0.34
											HC	3.33	0.032
		L. postcentral gyrus	-50	-24	30	65	0.000112*				pre-DREZ	-2.91	0.032
											post-DREZ	5.6	0.0066
											low-pain SCI	1.52	0.34
											HC	2.28	0.084
		L. precentral gyrus	-26	-24	66	56	0.000145*				pre-DREZ	-2.77	0.032
											post-DREZ	2.51	0.064
											low-pain SCI	0.06	0.97
											HC	1.41	0.25
		R. postcentral gyrus	30	-34	60	41	0.000131*				pre-DREZ	-3.76	0.028
											post-DREZ	1.82	0.15
											low-pain SCI	0.27	0.97
											HC	1.2	0.29
**Right Hippocampus**	**Group ANOVA**	medial pre and postcentral gyrus	18	-32	64	5723	<0.00001*	DREZ < Pain free	4.02	0.0012*			
								preop-DREZ < HC	7.51	0.000002*			
								low-pain SCI<HC	3.65	0.00055*			

								Preop < Postop	3.08	0.037			
											pre-DREZ	-5.09	0.0026*
											post-DREZ	-0.47	0.93
											low-pain SCI	0.24	0.81
											HC	5.67	0.00022*
		L. precentral gyrus	-44	-8	56	277	0.00001*	DREZ < Pain free	5.36	0.0003*			
								preop-DREZ < HC	5.69	0.00002*			
								low-pain SCI<HC	1.54	0.21			
								Preop < Postop	2.14	0.07			
											pre-DREZ	-4.9	0.0026*
											post-DREZ	0.09	0.93
											low-pain SCI	2.35	0.076
											HC	3.64	0.0039
		L. Heschl's	-40	-16	6	144	0.000041*	preop-DREZ < low-pain SCI	4.07	0.0012*			
								preop-DREZ < HC	6.2	0.000011*			
								low-pain SCI<HC	0.08	0.94			
								Preop < Postop	2.84	0.037			
											pre-DREZ	-3.38	0.011
											post-DREZ	1.91	0.29
											low-pain SCI	2.47	0.076
											HC	5.84	0.00022*
	**Pre>post DREZ**	R. Cerebellum crus 1	4	-80	-18	74	0.000017*				pre-DREZ	4.04	0.0049
											post-DREZ	-1.65	0.29
											low-pain SCI	-1.06	0.42
											HC	-0.54	0.6
		R. superior frontal gyrus	24	-2	70	61	0.000036*				pre-DREZ	-4.3	0.0049
											post-DREZ	0.82	0.44
											low-pain SCI	-0.85	0.42
											HC	1.13	0.57
**Left Hippocampus**	**Group ANOVA**	Medial pre and postcentral gyrus	18	-38	68	3156	<0.000001*	DREZ < Pain free	3.65	0.005			
								preop-DREZ < HC	8.3	<0.000001*			
								low-pain SCI<HC	5.82	0.000016*			
								Preop < Postop	5.52	0.0018*			
											pre-DREZ	-4.64	0.0024*
											post-DREZ	-0.56	0.59
											low-pain SCI	-1.04	0.34
											HC	7.49	0.000024*
		L. posterior temporal fusiform cortex	-36	-36	-12	132	0.000012*	preop-DREZ < low-pain SCI	2.31	0.036			
								preop-DREZ < HC	4.33	0.0004*			
								low-pain SCI<HC	6.38	0.00001*			
								Preop < Postop	2.06	0.082			
											pre-DREZ	8.79	0.0001*
											post-DREZ	8.73	0.0001*
											low-pain SCI	9.11	0.000079*
											HC	4.84	0.00052*
	**Pre>post DREZ**	Cerebellum crus 1	20	-76	-28	98	0.000006*				pre-DREZ	3.49	0.02
											post-DREZ	-3.96	0.011
											low-pain SCI	0.76	0.63
											HC	3.08	0.021

		Cerebellum crus 2	8	-88	-26	94	0.000034*				pre-DREZ	9.83	0.00001*
											post-DREZ	-1.45	0.19
											low-pain SCI	0.47	0.65
											HC	2.72	0.026
		R. Cerebellum 8	32	-60	-40	62	0.000025*				pre-DREZ	2.56	0.037
											post-DREZ	1.68	0.18
											low-pain SCI	1.15	0.58
											HC	0.15	0.88
		R. Hescl's gyrus	46	-8	-4	58	0.000152*				pre-DREZ	-2.71	0.037
											post-DREZ	6.85	0.00098*
											low-pain SCI	1.36	0.58
											HC	4.97	0.0017*
**PAG**	**Group ANOVA**	none											
	**Pre-post DREZ**	none											
**Thalamus**	**Group ANOVA**	none											
	**Pre-post DREZ**	none											

Follow up analyses of motor and somatosensory regions

Seed	Contrast	Resulting region	MNI x	MNI y	MNI z	cluster size	P_{FDR}	Posthoc contrast	T	P_{FDR}	Connectivity	T	P_{FDR}
**Superior motor intereffector region**													
	**Group ANOVA**	R. Hippocampus and Amygdala	24	-16	-20	361	0.000125*	DREZ < Pain free	1.47	0.2			
								preop-DREZ < HC	8.03	0.000001*			
								low-pain SCI<HC	5.28	0.00013*			
								Preop < Postop	1.44	0.24			
											pre-DREZ	-4.4	0.0052
											post-DREZ	-0.8	0.56
											low-pain SCI	-1.27	0.41
											HC	7.37	0.00007*
		Medial frontal cortex	-10	50	-20	324	0.00015*	DREZ < Pain free	-0.48	0.64			
								preop-DREZ < HC	6.08	0.000016*			
								low-pain SCI<HC	6.63	0.000016*			
								Preop < Postop	0.58	0.58			
											pre-DREZ	-3.2	0.015
											post-DREZ	-2.93	0.11
											low-pain SCI	-3.86	0.031
											HC	5.69	0.00035*
		L. Hippocampus	-32	-24	-18	157	0.01	DREZ < Pain free	2.53	0.039			
								preop-DREZ < HC	6.45	0.00005*			
								low-pain SCI<HC	3.67	0.0029*			
								Preop < Postop	1.73	0.21			
											pre-DREZ	-4.42	0.0052
											post-DREZ	-0.41	0.7
											low-pain SCI	-0.58	0.58
											HC	5.02	0.00065*
		R. caudate	0	6	0	117	0.028	DREZ < Pain free	2.94	0.027			
								preop-DREZ < HC	5.48	0.00004*			
								low-pain SCI<HC	2.82	0.0114			

								Preop < Postop	2.76	0.07			
											pre-DREZ	-4.18	0.0052
											post-DREZ	-1.64	0.36
											low-pain SCI	-1.33	0.41
											HC	2.82	0.021
		R. posterior supramarginal	68	-44	20	95	0.05	DREZ < Pain free	-3.6	0.014			
								preop-DREZ < HC	-5.29	0.00005*			
								low-pain SCI<HC	-1.1	0.28			
								Preop < Postop	-4.94	0.0084			
											pre-DREZ	5.13	0.0052
											post-DREZ	0.85	0.56
											low-pain SCI	0.77	0.58
											HC	-0.77	0.45
	**Pre-post DREZ**	R. cerebellum Crus 1 & 6	22	-70	-24	158	0.000075*				pre-DREZ	-4.22	0.0052
											post-DREZ	2.2	0.063
											low-pain SCI	-2.04	0.16
											HC	-2.13	0.11
		L. Thalmus	-10	-28	10	120	0.00037*				pre-DREZ	-2.67	0.032
											post-DREZ	3.22	0.028
											low-pain SCI	-0.52	0.82
											HC	-0.76	0.62
		R putamen, frontal orbital	20	6	-12	76	0.0048				pre-DREZ	-5.68	0.0015*
											post-DREZ	2.96	0.028
											low-pain SCI	-0.02	0.98
											HC	0.5	0.63
		L. precentral gyrus, SMA	-4	-16	52	57	0.015				pre-DREZ	13.75	0.00001*
											post-DREZ	5.75	0.0028*
											low-pain SCI	7.76	0.00044*
											HC	8.22	0.00002*
**Middle motor intereffector region**		L accumbens	-10	12	-8	209	0.004*	DREZ < Pain free	3.73	0.0022*			
								preop-DREZ < HC	6.7	0.00003*			
								low-pain SCI<HC	1.22	0.23			
								Preop < Postop	3.7	0.0076			
											pre-DREZ	-4.88	0.0018*
											post-DREZ	-3.17	0.025
											low-pain SCI	0.8	0.45
											HC	4.1	0.0018*
	**Pre-post DREZ**	R amygdala, hippocampus	20	8	-12	60	0.027				pre-DREZ	-3.27	0.018
											post-DREZ	4.74	0.0084
											low-pain SCI	0.7	0.74
											HC	3.46	0.021
		R. Superior parietal lobule	36	-52	48	62	0.041				pre-DREZ	2.81	0.026
											post-DREZ	-3.56	0.018
											low-pain SCI	0.35	0.74
											HC	1.11	0.29
		L- Parahippocampal gyrus	-22	-34	-10	46	0.041				pre-DREZ	-3.56	0.018

											post-DREZ	2.37	0.066
											low-pain SCI	-2.18	0.26
											HC	-1.65	0.25
		R. Thalamus and accumbens	4	8	-4	61	0.027				pre-DREZ	-6.7	0.0011*
											post-DREZ	0.08	0.93
											low-pain SCI	-0.6	0.74
											HC	1.35	0.27
**Inferior motor intereffector region**	**Group Anova**	None											
	**Pre-post DREZ**	None											
**Brodman area 1**	**Group Anova**	R. Middle fronbtal gyrus	32	14	32	160	0.018	DREZ < Pain free	7.84	0.00002*			
								preop-DREZ < HC	2.38	0.029			
								low-pain SCI<HC	-4.02	0.0008*			
								Preop < Postop	3.69	0.0078			
											pre-DREZ	-7.04	0.0002*
											post-DREZ	-1.05	0.33
											low-pain SCI	4.75	0.0021*
											HC	-1.3	0.22
	**Pre-post DREZ**	none											
**Brodman area 2**	**Group Anova**	none											
	**Pre-post DREZ**	None											
**Brodman area 3a**	**Group Anova**	Frontal Medial Cortex	8	38	-4	316	0.00033*	DREZ < Pain free	2.65	0.019			
								preop-DREZ < HC	6.54	0.000008*			
								low-pain SCI<HC	3.7	0.0066			
								Preop < Postop	3.66	0.008			
											pre-DREZ	-4.75	0.0028*
											post-DREZ	-0.67	0.57
											low-pain SCI	-1.66	0.28
											HC	3.89	0.0033*
		bilateral accumbens	4	6	-4	248	0.00093*	DREZ < Pain free	5.17	0.00057*			
								preop-DREZ < HC	6.09	0.000009*			
								low-pain SCI<HC	0.99	0.34			
								Preop < Postop	5	0.0031*			
											pre-DREZ	-6.85	0.00097*
											post-DREZ	-0.59	0.57
											low-pain SCI	0.18	0.86
											HC	1.65	0.13
		R. hippocampus	22	-10	-16	148	0.0085*	DREZ < Pain free	3.96	0.0024*			
								preop-DREZ < HC	6.1	0.000009*			
								low-pain SCI<HC	2.29	0.069			
								Preop < Postop	5.22	0.0031*			
											pre-DREZ	-3.9	0.0059
											post-DREZ	3.46	0.042
											low-pain SCI	2	0.28
											HC	5.41	0.00064*
		L. hippocampus	-24	-14	-16	146	0.0085*	DREZ < Pain free	3.85	0.0024*			
								preop-DREZ < HC	6.85	0.000008*			
								low-pain SCI<HC	1.88	0.1			
								Preop < Postop	4.6	0.0033*			
											pre-DREZ	-5.37	0.0021*
											post-DREZ	2.15	0.14
											low-pain SCI	1.34	0.3
											HC	5.15	0.00064*

	**Pre-post DREZ**	R. amygdala and accumbens	10	8	-8	389	<0.000000*				pre-DREZ	-7.59	0.00045*
											post-DREZ	4.38	0.0092
											low-pain SCI	0.57	0.86
											HC	1.06	0.53
		R. lateral ocippital cortex	24	-72	42	99	0.0012*				pre-DREZ	4.82	0.0032*
											post-DREZ	-2.57	0.046
											low-pain SCI	0.18	0.86
											HC	-1.35	0.53
		L hippocampus, amygdala	-26	-14	-10	86	0.002*				pre-DREZ	-3.8	0.0084
											post-DREZ	3.95	0.0092
											low-pain SCI	-0.18	0.86
											HC	0.65	0.53
		R. lateral ocippital cortex	22	-58	48	61	0.01				pre-DREZ	7.2	0.00045*
											post-DREZ	-4.2	0.0092
											low-pain SCI	1.44	0.49
											HC	0.96	0.53
		L. putamen	-24	4	-8	51	0.018				pre-DREZ	-2.52	0.04
											post-DREZ	2.14	0.07
											low-pain SCI	1.81	0.49
											HC	-0.66	0.53
**Brodman area 3b**	**Group Anova**	R. hippocampus	34	-22	-16		0.0021*	DREZ < Pain free	5.29	0.00023*			
								preop-DREZ < HC	6.5	0.000004*			
								low-pain SCI<HC	1.64	0.12			
								Preop < Postop	3.13	0.03			
											pre-DREZ	-4.77	0.0041*
											post-DREZ	1.43	0.2
											low-pain SCI	2.79	0.027
											HC	5.05	0.00037*
		L. hippocampus	-30	-16	-16		0.0038	DREZ < Pain free	4.37	0.00064*			
								preop-DREZ < HC	7.11	0.000003*			
								low-pain SCI<HC	1.98	0.12			
								Preop < Postop	2.72	0.03			
											pre-DREZ	-3.61	0.0086
											post-DREZ	1.52	0.2
											low-pain SCI	2.81	0.027
											HC	6.91	0.000051*
	**Pre-post DREZ**	R amygdala	6	4	-10	131	0.00032*				pre-DREZ	-7.01	0.00048*
											post-DREZ	2.63	0.068
											low-pain SCI	-1.23	0.34
											HC	-0.14	0.89
		R superior parietal lobule	18	-54	46	74	0.0079				pre-DREZ	6.87	0.00048*
											post-DREZ	-3.56	0.038
											low-pain SCI	2.45	0.088
											HC	0.95	0.48
		R. lateral ocipital cortex	26	-72	42	53	0.027				pre-DREZ	3.76	0.0071
											post-DREZ	-1.66	0.19
											low-pain SCI	0.9	0.4
											HC	-1.29	0.48
		R. frontal orbital cortex	36	30	-14	50	0.027				pre-DREZ	-6.42	0.00048*
											post-DREZ	1.35	0.21
											low-pain SCI	-2.98	0.083
											HC	-0.97	0.48

Supplemental Table 1. Complete results for all seed regions, including group differences between healthy controls (HC), persons with spinal cord injury and low pain (low-pain SCI) and persons scheduled for dorsal root entry zone surgery (pre-op DREZ). Post-hoc-contrasts, as well as the T-score of connectivity in each group is reported for completeness. Follow-up analyses of motor and somatosensory regions are also reported. Montreal Neurological Institute x,y.z coordinates (MNI_{xyz}), probability value after correction for False Discovery Rate (P_{FDR}), Periaqueductal gray matter (PAG)

Supplemental references

1. Gordon EM, Chauvin RJ, Van AN, et al. A somato-cognitive action network alternates with effector regions in motor cortex. *Nature*. May 2023;617(7960):351-359. doi:10.1038/s41586-023-05964-2
2. Geyer S, Schormann T, Mohlberg H, Zilles K. Areas 3a, 3b, and 1 of human primary somatosensory cortex. Part 2. Spatial normalization to standard anatomical space. *NeuroImage*. Jun 2000;11(6 Pt 1):684-96. doi:10.1006/nimg.2000.0548
3. Friston KJ, Holmes AP, Worsley KJ, Poline J-P, Frith CD, Frackowiak RSJ. Statistical parametric maps in functional imaging: A general linear approach. *Human brain mapping*. 1994;2(4):189-210. doi:https://doi.org/10.1002/hbm.460020402
4. Ashburner J, Friston KJ. Voxel-based morphometry--the methods. Research Support, Non-U.S. Gov't
Review. *NeuroImage*. Jun 2000;11(6 Pt 1):805-21. doi:10.1006/nimg.2000.0582
5. Ashburner J. A fast diffeomorphic image registration algorithm. *NeuroImage*. Oct 15 2007;38(1):95-113. doi:10.1016/j.neuroimage.2007.07.007
6. Dale AM, Fischl B, Sereno MI. Cortical surface-based analysis. I. Segmentation and surface reconstruction. *NeuroImage*. Feb 1999;9(2):179-94. doi:10.1006/nimg.1998.0395
7. Reuter M, Schmansky NJ, Rosas HD, Fischl B. Within-subject template estimation for unbiased longitudinal image analysis. *NeuroImage*. Jul 16 2012;61(4):1402-18. doi:10.1016/j.neuroimage.2012.02.084
8. Tournier JD, Smith R, Raffelt D, et al. MRtrix3: A fast, flexible and open software framework for medical image processing and visualisation. *NeuroImage*. Nov 15 2019;202:116137. doi:10.1016/j.neuroimage.2019.116137
9. Smith SM, Jenkinson M, Woolrich MW, et al. Advances in functional and structural MR image analysis and implementation as FSL. *NeuroImage*. 2004;23 Suppl 1:S208-19. doi:10.1016/j.neuroimage.2004.07.051
10. Yendiki A, Panneck P, Srinivasan P, et al. Automated probabilistic reconstruction of white-matter pathways in health and disease using an atlas of the underlying anatomy. *Front Neuroinform*. 2011;5:23. doi:10.3389/fninf.2011.00023
11. Jones DK, Travis AR, Eden G, Pierpaoli C, Basser PJ. PASTA: pointwise assessment of streamline tractography attributes. *Magnetic resonance in medicine*. Jun 2005;53(6):1462-7. doi:10.1002/mrm.20484
12. Woolrich MW, Jbabdi S, Patenaude B, et al. Bayesian analysis of neuroimaging data in FSL. *NeuroImage*. Mar 2009;45(1 Suppl):S173-86. doi:10.1016/j.neuroimage.2008.10.055
13. Jenkinson M, Beckmann CF, Behrens TE, Woolrich MW, Smith SM. Fsl. *NeuroImage*. Aug 15 2012;62(2):782-90. doi:10.1016/j.neuroimage.2011.09.015
14. Cordero-Grande L, Christiaens D, Hutter J, Price AN, Hajnal JV. Complex diffusion-weighted image estimation via matrix recovery under general noise models. *NeuroImage*. Oct 15 2019;200:391-404. doi:10.1016/j.neuroimage.2019.06.039
15. Veraart J, Novikov DS, Christiaens D, Ades-Aron B, Sijbers J, Fieremans E. Denoising of diffusion MRI using random matrix theory. *NeuroImage*. Nov 15 2016;142:394-406. doi:10.1016/j.neuroimage.2016.08.016

16. Kellner E, Dhital B, Kiselev VG, Reisert M. Gibbs-ringing artifact removal based on local subvoxel-shifts. *Magnetic resonance in medicine*. Nov 2016;76(5):1574-1581. doi:10.1002/mrm.26054
17. Andersson JLR, Graham MS, Drobnjak I, Zhang H, Filippini N, Bastiani M. Towards a comprehensive framework for movement and distortion correction of diffusion MR images: Within volume movement. *NeuroImage*. May 15 2017;152:450-466. doi:10.1016/j.neuroimage.2017.02.085
18. Andersson JLR, Sotiropoulos SN. An integrated approach to correction for off-resonance effects and subject movement in diffusion MR imaging. *NeuroImage*. Jan 15 2016;125:1063-1078. doi:10.1016/j.neuroimage.2015.10.019
19. Bastiani M, Cottaar M, Fitzgibbon SP, et al. Automated quality control for within and between studies diffusion MRI data using a non-parametric framework for movement and distortion correction. *NeuroImage*. Jan 1 2019;184:801-812. doi:10.1016/j.neuroimage.2018.09.073
20. Smith SM. Fast robust automated brain extraction. *Human brain mapping*. Nov 2002;17(3):143-55. doi:10.1002/hbm.10062
21. Smith SM, Jenkinson M, Johansen-Berg H, et al. Tract-based spatial statistics: voxelwise analysis of multi-subject diffusion data. *NeuroImage*. Jul 15 2006;31(4):1487-505. doi:10.1016/j.neuroimage.2006.02.024
22. Winkler AM, Ridgway GR, Webster MA, Smith SM, Nichols TE. Permutation inference for the general linear model. *NeuroImage*. May 15 2014;92(100):381-97. doi:10.1016/j.neuroimage.2014.01.060
23. Maffei C, Lee C, Planich M, et al. Using diffusion MRI data acquired with ultra-high gradient strength to improve tractography in routine-quality data. *NeuroImage*. Dec 15 2021;245:118706. doi:10.1016/j.neuroimage.2021.118706
24. Greve DN, Fischl B. Accurate and robust brain image alignment using boundary-based registration. *NeuroImage*. Oct 15 2009;48(1):63-72. doi:10.1016/j.neuroimage.2009.06.060
25. Behrens TE, Woolrich MW, Jenkinson M, et al. Characterization and propagation of uncertainty in diffusion-weighted MR imaging. *Magnetic resonance in medicine*. Nov 2003;50(5):1077-88. doi:10.1002/mrm.10609
26. Sotiropoulos SN, Hernandez-Fernandez M, Vu AT, et al. Fusion in diffusion MRI for improved fibre orientation estimation: An application to the 3T and 7T data of the Human Connectome Project. *NeuroImage*. Jul 1 2016;134:396-409. doi:10.1016/j.neuroimage.2016.04.014
27. Jbabdi S, Sotiropoulos SN, Savio AM, Grana M, Behrens TE. Model-based analysis of multishell diffusion MR data for tractography: how to get over fitting problems. *Magnetic resonance in medicine*. Dec 2012;68(6):1846-55. doi:10.1002/mrm.24204
28. Behrens TE, Berg HJ, Jbabdi S, Rushworth MF, Woolrich MW. Probabilistic diffusion tractography with multiple fibre orientations: What can we gain? *NeuroImage*. Jan 1 2007;34(1):144-55. doi:10.1016/j.neuroimage.2006.09.018
